# Secretome profiling of *Cryptococcus neoformans* reveals regulation of a subset of virulence-associated proteins and potential biomarkers by protein kinase A

**DOI:** 10.1186/s12866-015-0532-3

**Published:** 2015-10-09

**Authors:** Jennifer M. H. Geddes, Daniel Croll, Mélissa Caza, Nikolay Stoynov, Leonard J. Foster, James W. Kronstad

**Affiliations:** Michael Smith Laboratories, University of British Columbia, Vancouver, BC V6T 1Z4 Canada; Department of Microbiology and Immunology, University of British Columbia, Vancouver, BC V6T 1Z4 Canada; Centre for High-Throughput Biology, University of British Columbia, Vancouver, BC V6T 1Z4 Canada

**Keywords:** Quantitative proteomics, Fungal pathogenesis, Secretome, PKA, Virulence factors, Biomarkers, Multiple reaction monitoring

## Abstract

**Background:**

The pathogenic yeast *Cryptococcus neoformans* causes life-threatening meningoencephalitis in individuals suffering from HIV/AIDS. The cyclic-AMP/protein kinase A (PKA) signal transduction pathway regulates the production of extracellular virulence factors in *C. neoformans,* but the influence of the pathway on the secretome has not been investigated. In this study, we performed quantitative proteomics using galactose-inducible and glucose-repressible expression of the *PKA1* gene encoding the catalytic subunit of PKA to identify regulated proteins in the secretome.

**Methods:**

The proteins in the supernatants of cultures of *C. neoformans* were precipitated and identified using liquid chromatography-coupled tandem mass spectrometry. We also employed multiple reaction monitoring in a targeted approach to identify fungal proteins in samples from macrophages after phagocytosis of *C. neoformans* cells, as well as from the blood and bronchoalveolar fluid of infected mice.

**Results:**

We identified 61 secreted proteins and found that changes in *PKA1* expression influenced the extracellular abundance of five proteins, including the Cig1 and Aph1 proteins with known roles in virulence. We also observed a change in the secretome profile upon induction of Pka1 from proteins primarily involved in catabolic and metabolic processes to an expanded set that included proteins for translational regulation and the response to stress. We further characterized the secretome data using enrichment analysis and by predicting conventional versus non-conventional secretion. Targeted proteomics of the Pka1-regulated proteins allowed us to identify the secreted proteins in lysates of phagocytic cells containing *C. neoformans*, and in samples from infected mice. This analysis also revealed that modulation of *PKA1* expression influences the intracellular survival of cryptococcal cells upon phagocytosis.

**Conclusions:**

Overall, we found that the cAMP/PKA pathway regulates specific components of the secretome including proteins that affect the virulence of *C. neoformans*. The detection of secreted cryptococcal proteins from infected phagocytic cells and tissue samples suggests their potential utility as biomarkers of infection. The proteomics data are available via ProteomeXchange with identifiers PXD002731 and PASS00736.

**Electronic supplementary material:**

The online version of this article (doi:10.1186/s12866-015-0532-3) contains supplementary material, which is available to authorized users.

## Background

*Cryptococcus neoformans* is an opportunistic, yeast-like fungus that is a significant threat to immunocompromised individuals such as patients with HIV/AIDS [1, 2]. The ability of *C. neoformans* to cause disease depends on the production of virulence factors including a polysaccharide capsule, melanin deposition in the cell wall, the ability to grow at 37 °C, and the secretion of extracellular enzymes [3–8]. Extracellular enzymes with roles in virulence include phospholipases, which hydrolyze ester bonds and aid in the degradation and destabilization of host cell membranes and cell lysis, and urease, which hydrolyzes urea to ammonia and carbamate, inducing a localized increase in pH [9–12]. Proteinases may also cause tissue damage, provide nutrients to the pathogen and facilitate migration to the central nervous system [13–15]. In general, the secretion of extracellular enzymes is important for fungal survival within the host but a comprehensive investigation of the secretome and its regulation by the cyclic-AMP/Protein Kinase A (PKA) signal transduction pathway has not been performed for *C. neoformans.*

The cAMP/PKA pathway regulates capsule production, melanin formation, mating, and virulence in *C. neoformans* [16–20]*.* Components of the pathway include a Gα protein (Gpa1), adenylyl cyclase (Cac1), adenylyl cyclase-associated protein (Aca1), a candidate receptor (Gpr4), phosphodiesterases (Pde1 and Pde2), and the PKA catalytic (Pka1, Pka2) and regulatory (Pkr1) subunits. In response to environmental signals, including exogenous methionine and nutrient starvation, the G-protein coupled receptor (GPCR), Gpr4 undergoes a conformational change to activate Cacl and subsequently stimulate the production of cAMP. Mutations in genes encoding the Gpa1, Cac1, Aca1, and Pka1 proteins result in reduced formation of capsule and melanin, as well as sterility and attenuated virulence in a mouse model of cryptococcosis [16, 21]. In particular, Pka1 is a key regulator of virulence in *C. neoformans*. In contrast, disruption of the gene encoding Pkr1 results in enlargement of the capsule and hypervirulence [17].

Previous transcriptional profiling experiments compared a wild-type strain with *pka1*Δ and *pkr1*Δ mutants of *C. neoformans*, and identified differences in transcript levels for genes related to cell wall synthesis, transport (e.g., iron uptake), the tricarboxylic acid cycle, and glycolysis [22]. Differential expression patterns were also observed for genes encoding ribosomal proteins, stress and chaperone functions, secretory pathway components and phospholipid biosynthetic enzymes. Specifically, loss of *PKA1* influenced the expression of genes involved in secretion, and Pka1 was hypothesized to influence capsule formation by regulating expression of secretory pathway components that control the export of capsular polysaccharide to the cell surface. Additionally, the secretion inhibitors brefeldin A, nocodazole, monensin, and NEM reduced capsule size, a phenotype similar to that observed in a *pka1* mutant [22]. In general, the mechanisms and components required for the export of capsule polysaccharide and other virulence factors in *C. neoformans* are poorly understood. Beyond the role of PKA, other studies have examined exocytosis functions (Sec6, Sec14), the secretion of phospholipases, and the involvement of extracellular vesicles [23–28]. Additionally, O’Meara et al. (2010) recently demonstrated that PKA influences capsule attachment via phosphorylation of the pH-responsive transcription factor Rim101, a key regulator of cell wall functions.

The role of PKA in secretion in *C. neoformans* has also been examined with strains carrying galactose-inducible and glucose-repressible versions of *PKA1* and *PKR1* constructed by inserting the *GAL7* promoter upstream of the genes [29]. Elevated Pka1 activity, stimulated by growth of the *P*_*GAL7*_*::PKA1* strain in galactose-containing media, was found to influence capsule thickness, cell size, ploidy, and vacuole enlargement [29]. The authors also showed that Pka1 activity was required for wild-type levels of melanization and laccase activity, and influenced the correct localization of laccase. The ability to regulate expression of *PKA1* and, subsequently, the activity of Pka1, is a powerful tool for investigating the mechanisms of its influence on the secretion of virulence factors and secretory pathway components.

In this study, we used the strain with galactose-inducible and glucose-repressible expression of *PKA1* to investigate the influence of Pka1 on the secretome using quantitative proteomics. We identified 61 different secreted proteins and found that Pka1 regulated the extracellular abundance of five. These proteins included three enzymes (α-amylase, acid phosphatase, and glyoxal oxidase), the Cig1 protein (cytokine-inducing glycoprotein) associated with virulence and heme uptake, and a novel protein containing a carbohydrate-binding domain (CNAG_05312). We also observed a change in the secretome profile under Pka1-inducing conditions from proteins involved primarily in catabolic and metabolic processes to an expanded set that included proteins for translational regulation and the response to stress. Enrichment analysis of our Pka1-influenced secretome data compared to the whole genome showed over-representation of genes associated with a broad spectrum of processes including metabolic and catabolic processing. Although no enrichment was observed between our secretome data and the Fungal Secretome KnowledgeBase (FunSecKB), a comparison of GO terms between the data sets showed the majority of our identified proteins to be represented in the FunSecKB. Next, we exploited our secretome data using a targeted proteomics approach to identify potential biomarkers of cryptococcal infection. Multiple Reaction Monitoring (MRM) in the presence of stable isotope dilutions (SID) allows for identification and quantification of specific peptides in a sample. Specifically, we were able to identify Pka1-regulated proteins of *C. neoformans* in host samples including blood, bronchoalveolar lavage fluid, and infected macrophage lysates. Overall, our study reveals that the cAMP/PKA pathway regulates specific components of the secretome including the Cig1 and Aph1 proteins that contribute to virulence in *C. neoformans*.

## Results

### Control of *PKA1* expression results in a change of the protein secretion profile

**Fig. 1:**
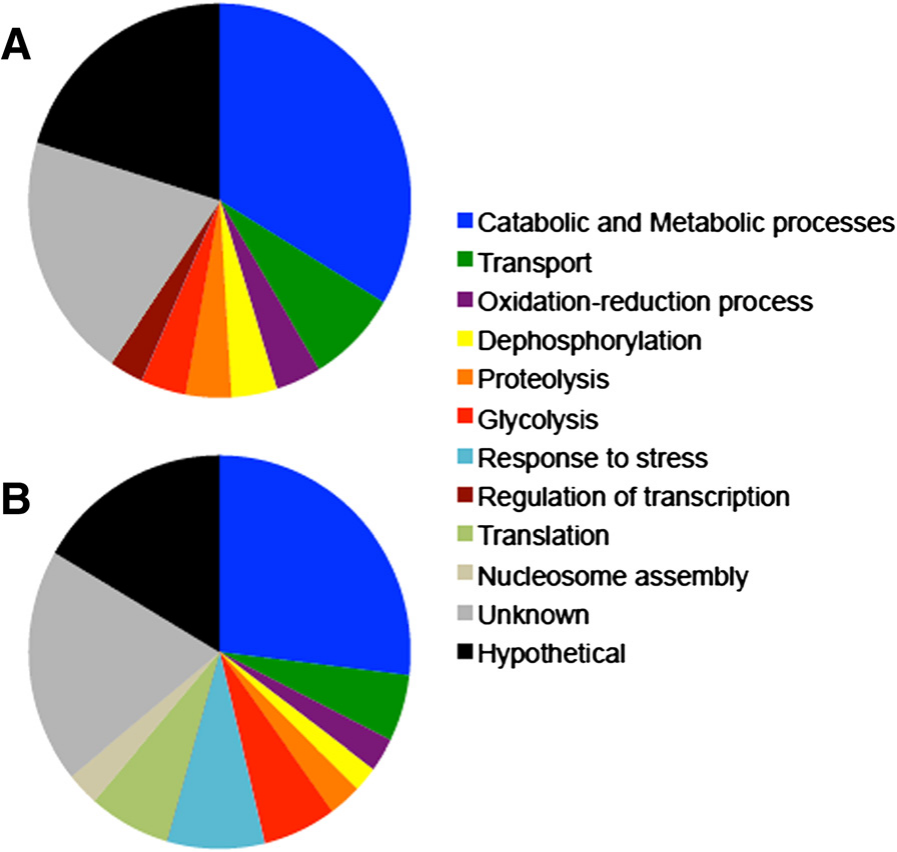
Quantitative proteomic analysis of the *C. neoformans* secretome over the course of all time-points (16, 48, 72, and 120 hpi) under (**a**) Pka1-repressed (glucose) and (**b**) Pka1-induced (galactose) conditions. Identified proteins were grouped according to GO terms associated with their biological classifications. GO term classification was performed on unique proteins identified under either Pka1-repressed or Pka1-induced conditions to highlight the overall influence of Pka1 regulation on the secretome profile

Given the virulence defect of a *pka1* mutant, we hypothesized that Pka1 influences the secretion of proteins associated with the virulence and survival of *C. neoformans* in the host. To test this idea, we quantitatively identified proteins secreted by *C. neoformans* in the context of regulated expression of *PKA1*. For our initial analysis, we collected supernatant cultures of WT and *P*_*GAL7*_*::PKA1* strains grown under Pka1-repressed (glucose) and Pka1-induced (galactose) conditions at 16, 48, 72, and 120 h post-inoculation (hpi), and analyzed the samples using quantitative mass spectrometry. The analysis of these supernatant samples resulted in the identification of 164 (54 quantifiable) and 207 (83 quantifiable) proteins under Pka1-repressed and Pka1-induced conditions, respectively (see Additional file 1: Table S1; Additional file 2: Table S2). As shown in Table 1, 23 proteins were identified and quantified under Pka1-repressed and Pka1-induced conditions at the specified time-points. We found that none of the changes in protein abundance between the two conditions were statistically significant (*p* > 0.05) and therefore, concluded that Pka1 did not influence the abundance of any of the observed proteins under the conditions tested. However, upon comparison of the unique proteins identified under either Pka1-repressed or Pka1-induced conditions, using Gene Ontology (GO) term biological classifications at all time points, we were able to observe overall changes in the secretome profiles under the influence of Pka1 (Fig. 1). Additional differentially expressed proteins may be present in the samples, but we were unable to measure their abundance and they were therefore not included for further analysis. Under Pka1-repressed conditions, the majority of secreted proteins were associated with catabolic and metabolic (33 %), unknown (20 %), and hypothetical (20 %) processes (totaling 73 %), with additional proteins associated with transport (8 %), oxidation-reduction processes (4 %), dephosphorylation (4 %), proteolysis (4 %), glycolysis (4 %), and regulation of transcription (3 %). Conversely, a change in the secretome profile was observed under the Pka1-induction condition. Here, we again observed the majority of proteins to be associated with catabolic and metabolic (26 %), unknown (19 %), and hypothetical (17 %) processes (totaling 62 %). A slight decline was found for proteins associated with transport (from 8 to 6 %), oxidation-reduction processes (from 4 to 3 %), dephosphorylation (from 4 to 2 %), proteolysis (from 4 to 3 %), and regulation of transcription (from 3 to 0 %). However, a greater emphasis was found for proteins associated with glycolysis (from 4 to 6 %), response to stress (from 0 to 8 %), translation (from 0 to 7 %), and nucleosome assembly (from 0 to 3 %). Although, our secretome analysis at specific times did not identify Pka1-regulated proteins, a change toward the secretion of proteins for glycolysis, translational regulation, nucleosome assembly, and the response to stress was observed upon induction of *PKA1* expression.

**Table 1:** Proteins identified in the secretome of *C. neoformans* collected at 16, 48, 72, and 120 hpi grown in Pka1-repressed (glucose-containing medium) and Pka1-induced (galactose-containing medium) conditions

GO categories^a^	Accession number	Protein Name		Fold change^b*^		Std. Dev.	
			Time point	Pka1-repression	Pka1-induction	Pka1-repression	Pka1-induction
Carbohydrate catabolic process							
	CNAG_02189	α-Amylase	16 hpi	0.298	1.096	0.314	1.270
GTP catabolic process							
	CNAG_06125	Translation elongation factor 1 α	16 hpi	0.135	0.379	0.145	0.517
Carbohydrate metabolic process							
	CNAG_01239	Chitin deacetylase	16 hpi	0.906	1.350	0.310	0.515
	CNAG_04245	Chitinase	16 hpi	0.448	0.286	0.007	0.296
			48 hpi	0.826	0.603	1.019	0.017
	CNAG_06501	1,3-β-glucanosyltransferase	16 hpi	0.573	0.896	0.383	0.169
Transmembrane transport							
	CNAG_02974	Voltage-dependent ion-selective channel	16 hpi	0.223	0.466	0.089	0.645
Oxidation-reduction process							
	CNAG_03465	Laccase	16 hpi	0.637	0.870	0.651	1.038
Unknown/Unclassified							
	CNAG_02030	Glyoxal oxidase	16 hpi	0.278	0.600	0.324	0.672
			48 hpi	0.242	0.350	0.063	0.144
	CNAG_06267	Rds1 protein	16 hpi	0.336	0.756	0.303	0.222
			120 hpi	0.860	5.106	0.444	4.588
	CNAG_00776	Immunoreactive mannoprotein MP88	16 hpi	0.627	0.994	0.214	1.193
	CNAG_02864	Predicted protein	16 hpi	0.346	0.259	0.056	0.183
	CNAG_04753	Lactonohydrolase	16 hpi	1.031	0.751	0.631	0.413
			48 hpi	1.250	1.055	0.996	0.546
Hypothetical							
	CNAG_00587	Hypothetical protein	16 hpi	1.275	1.620	0.018	0.371
	CNAG_01047	Hypothetical protein	16 hpi	0.279	0.471	0.070	0.272
			72 hpi	0.928	0.719	1.034	0.432
	CNAG_03492	Hypothetical protein	16 hpi	0.506	0.799	0.400	0.030
			72 hpi	0.640	1.180	0.199	0.431
	CNAG_05893	Hypothetical protein	48 hpi	1.609	1.676	1.380	1.639
			120 hpi	1.641	1.413	0.953	0.348

^a^Proteins were categorized based on GO terms associated with their biological classification

^b^Fold change is reported as the average quantification for *P*_*GAL7*_ *::PKA1* vs. WT, ± standard deviation

^*^Statistical analysis was performed using a Student’s *t-test* between the conditions. None of the comparisons resulted in a significant difference in protein abundance (*p-value* > 0.05)

### Identification of secreted proteins regulated by Pka1

**Fig. 2:**
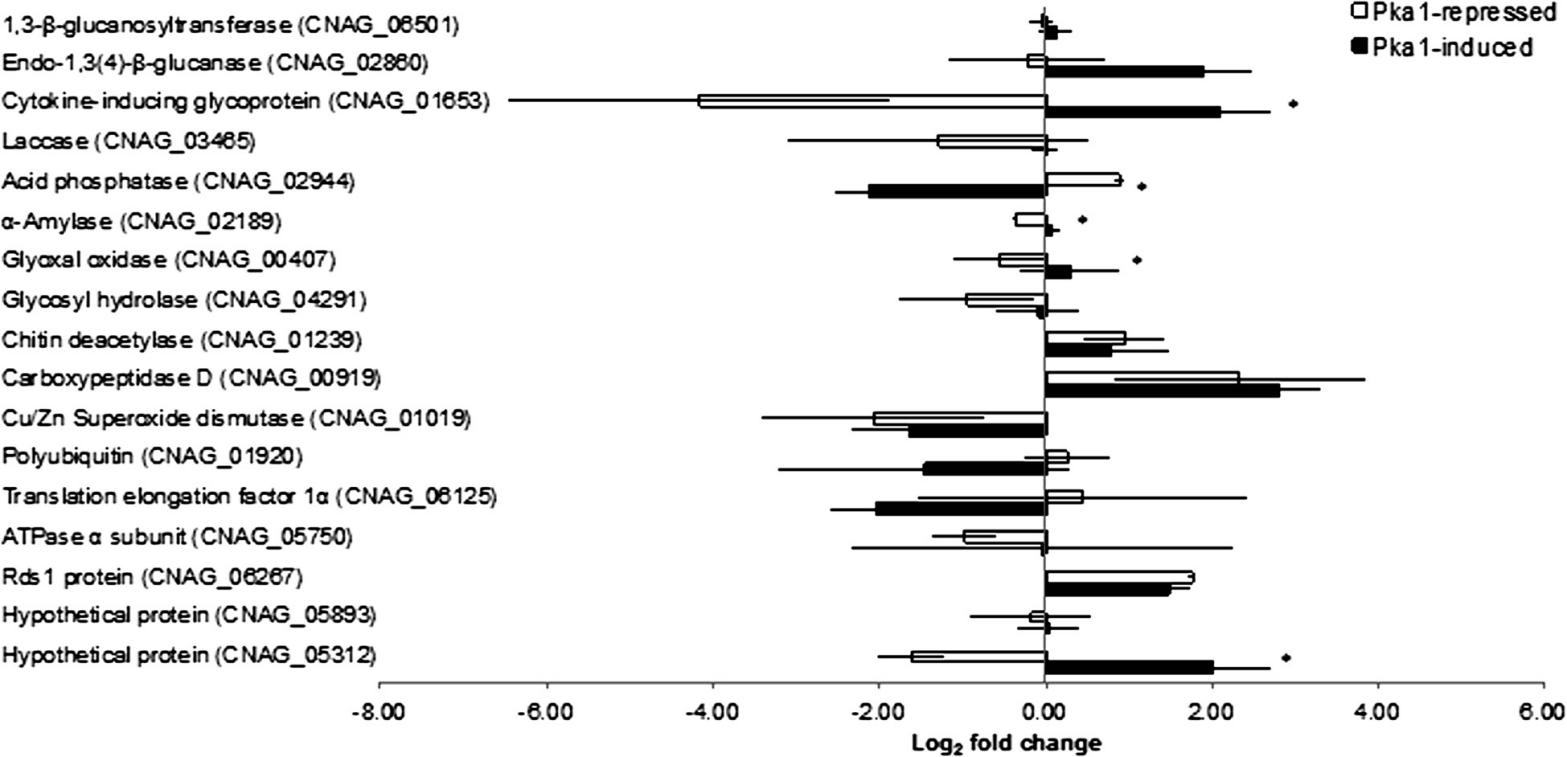
Quantitative proteomic analysis of the *C. neoformans* secretome under Pka1-repressed (glucose) and Pka1-induced (galactose) conditions. The secreted proteins were identified and quantified by LC-MS/MS in the *P*_*GAL7*_ *::PKA1* strain compared to WT, and the log_2_ of relative fold changes are indicated. Fold change is reported as the average log_2_ quantification for *P*_*GAL7*_ *::PKA1* vs. WT, ± standard deviation. Statistical analysis was performed using a Student’s *t-test* (*p-value* < 0.05), between conditions

Given that we identified secreted proteins from strains with modulated Pka1 activity, but did not observe any proteins whose abundance was directly regulated by Pka1, we extended our analysis to examine protein secretion at an intermediate time point of 96 hpi, and we used an alternative, less stringent method for protein precipitation (EtOH/acetate). We chose an end-point collection time of 96 hpi based on our coverage of a range of other time points in the previous analysis and because this time was sufficient for the culture to reach stationary phase and to accumulate proteins in the extracellular environment. Additionally, because we did not observe changes in protein abundance under regulation of Pka1 following the time-point analysis, we used the alternative protein precipitation method in an attempt to obtain a more comprehensive view of the secretome. We collected supernatant cultures of WT and *P*_*GAL7*_*::PKA1* strains grown under Pka1-repressed (glucose) and Pka1-induced (galactose) conditions at 96 hpi and analyzed the samples using quantitative mass spectrometry. Similar trends in protein abundance were observed for the majority of proteins in both experimental approaches (EtOH and TCA/acetone precipitation) (see Additional file 3: Table S3) [30]. Although the variability of the time-point analysis was relatively high, the reproducibility observed from the end-point analysis suggested that collecting the samples at different time-points impacted the protein abundance and contributed to the observed variability. This impact may be associated with culture sampling, as well as changes in capsule production during the early- to mid-log growth phases of the fungal cultures [29]. We identified 61 proteins under Pka1-repressed conditions of which 34 were successfully dimethyl-labeled and quantified (Table 2; see Additional file 4: Table S4). These 34 proteins covered a broad spectrum of biological classifications (17 categories) for GO terms, including proteins associated with catabolic and metabolic processes, ubiquitination, transport, dephosphorylation, glycolysis, oxidation-reduction, translation, proteolysis, and the response to stress. Under Pka1-induced conditions, we identified 38 proteins, of which 21 were successfully dimethyl-labeled and quantified (Table 3; see Additional file 5: Table S5). These 21 proteins covered 11 biological classifications for GO terms and included proteins associated with catabolic and metabolic processes, along with ubiquitination, transport, dephosphorylation, oxidation-reduction, proteolysis, and the response to stress. In total, 17 proteins were present under both Pka1-repressed and Pka1-induced conditions. A comparison of changes in abundance under Pka1-repressed and Pka1-induced conditions of these 17 proteins revealed that five showed statistically significant differences (*p-value* < 0.05, Student’s *t-test*) in abundance in response to regulation of Pka1 (Fig. 2). We concluded that the extracellular abundance of these five proteins was influenced by PKA and we focused our subsequent analysis on these proteins. Under Pka1-induced conditions, a cytokine-inducing glycoprotein (Cig1), an α-amylase, a glyoxal oxidase, and a novel protein (CNAG_05312) each showed an increase in abundance, whereas an acid phosphatase (Aph1) showed a decrease in abundance. Taken together, these findings suggest that Pka1 regulates the extracellular abundance of specific proteins secreted by *C. neoformans*.

**Table 2:** Proteins identified in the secretome of *C. neoformans* collected at 96 hpi from cells grown in Pka1-repressed (glucose-containing medium) conditions

GO categories^a^	Accession number	Protein name	Fold change^b^	Std. Dev.
Carbohydrate catabolic process				
	CNAG_02189	α-Amylase	0.777	0.019
GTP catabolic process				
	CNAG_06125	Translation elongation factor 1 α	2.062	2.181
Carbohydrate metabolic process				
	CNAG_02860	Endo-1,3(4)-β-glucanase	0.947	0.583
	CNAG_06501	1,3-β-glucanosyltransferase	0.968	0.100
	CNAG_05799	Chitin deacetylase	1.439	0.775
	CNAG_06291	Deacetylase	1.464	0.778
	CNAG_01239	Chitin deacetylase	2.014	0.584
Cellular carbohydrate metabolic process				
	CNAG_03225	Malate dehydrogenase	0.472	0.546
Pentose-phosphate pathway				
	CNAG_07561	Phosphogluconate dehydrogenase	>10	>10
Protein ubiquitination				
	CNAG_01920	Polyubiquitin	1.261	0.396
ATP hydrolysis coupled proton transport				
	CNAG_05918	F0F1 ATP synthase subunit β	0.451	0.249
	CNAG_05750	ATPase α subunit	0.604	0.387
Transmembrane transport				
	CNAG_06101	Eukaryotic ADP/ATP carrier	7.524	9.753
Methionine biosynthetic process				
	CNAG_01890	5-methyltetrahydropteroyltriglutamate-homocysteine S-methyltransferase	0.778	0.327
Dephosphorylation				
	CNAG_02944	Acid phosphatase	1.862	0.083
Glycolysis				
	CNAG_03072	Phosphopyruvate hydratase	3.093	3.377
Oxidation-reduction process				
	CNAG_01019	Cu/Zn superoxide dismutase	0.302	0.161
	CNAG_03465	Laccase	0.718	0.668
Proteolysis				
	CNAG_00919	Carboxypeptidase D	4.664	5.261
Response to stress				
	CNAG_01727	Hsc70-4	1.883	2.094
Translation				
	CNAG_06095	Ribosomal protein L13	0.147	0.057
Unknown/Unclassified				
	CNAG_01653	Cytokine-inducing glycoprotein	0.134	0.158
	CNAG_00407	Glyoxal oxidase	0.719	0.230
	CNAG_04291	Glycosyl-hydrolase	0.583	0.287
	CNAG_02030	Glyoxal oxidase	0.513	0.109
	CNAG_06267	Rds1 protein	3.409	0.106
Hypothetical				
	CNAG_05312	Conserved hypothetical protein	0.341	0.100
	CNAG_03007	Conserved hypothetical protein	0.518	0.649
	CNAG_01562	Conserved hypothetical protein	0.942	0.194
	CNAG_05893	Conserved hypothetical protein	0.991	0.487
	CNAG_01047	Conserved hypothetical protein	6.714	6.494
	CNAG_00588	Conserved hypothetical protein	>10	>10
	CNAG_03223	Conserved hypothetical protein	>10	>10
	CNAG_00586	Conserved hypothetical protein	>10	>10

^a^Proteins were categorized based on GO terms associated with their biological classification

^b^Fold change is reported as the average quantification for *P*_*GAL7*_ *::PKA1* vs. WT, ± standard deviation

**Table 3:** Proteins identified in the secretome of *C. neoformans* collected at 96 hpi from cells grown in Pka1-induced (galactose-containing medium) conditions

GO categories^a^	Accession number	Protein name	Fold change^b^	Std. Dev.
Carbohydrate catabolic process				
	CNAG_02189	α-Amylase	1.061	0.080
GTP catabolic process				
	CNAG_06125	Translation elongation factor 1 α	0.462	0.555
Carbohydrate metabolic process				
	CNAG_04245	Chitinase	0.621	0.562
	CNAG_06501	1,3-β-glucanosyltransferase	1.101	0.140
	CNAG_01239	Chitin deacetylase	2.659	1.996
	CNAG_02860	Endo-1,3(4)-β-glucanase	3.210	1.665
Protein ubiquitination				
	CNAG_01920	Polyubiquitin	0.509	0.501
ATP hydrolysis coupled proton transport				
	CNAG_05750	ATPase α subunit	1.785	1.765
Dephosphorylation				
	CNAG_02944	Acid phosphatase	0.233	0.061
Oxidation-reduction process				
	CNAG_01019	Cu/Zn superoxide dismutase	0.350	0.142
	CNAG_03465	Laccase	1.000	0.107
Proteolysis				
	CNAG_00919	Carboxypeptidase D	5.770	3.708
Response to stress				
	CNAG_01750	Chaperone	>10	>10
Unknown/Unclassified				
	CNAG_04291	Glycosyl-hydrolase	0.989	0.959
	CNAG_00407	Glyoxal oxidase	1.209	0.648
	CNAG_06267	Rds1 protein	2.834	0.518
	CNAG_01653	Cytokine-inducing glycoprotein	2.951	2.753
	CNAG_04753	Lactonohydrolase	>10	>10
Hypothetical				
	CNAG_06109	Conserved hypothetical protein	0.463	0.431
	CNAG_05893	Conserved hypothetical protein	1.062	0.307
	CNAG_05312	Conserved hypothetical protein	3.737	2.342

^a^Proteins were categorized based on GO terms associated with their biological classification

^b^Fold change is reported as the average quantification for *P*_*GAL7*_ *::PKA1* vs. WT, ± standard deviation

### Gene ontology analyses of the secretome revealed enrichment of proteins associated with metabolic and catabolic processes

**Fig. 3:**
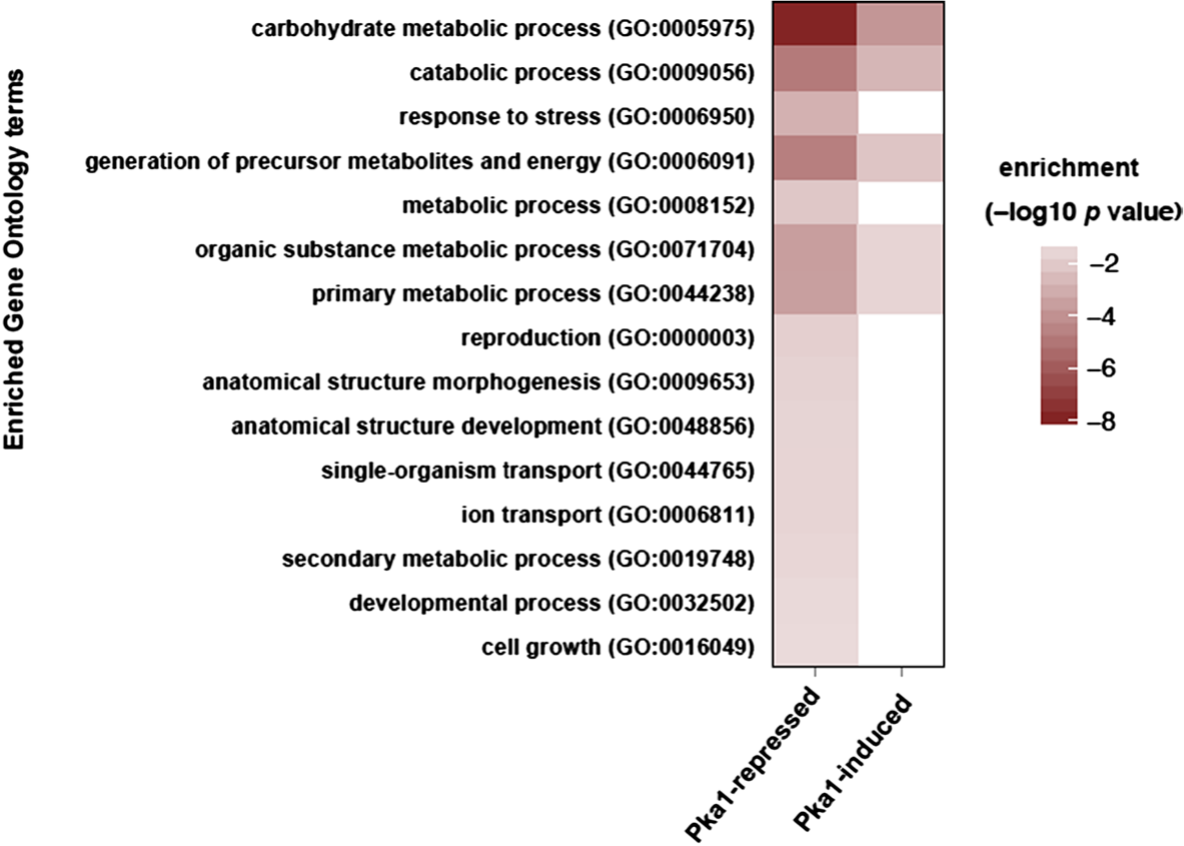
Enrichment of genes represented in the secretome analysis of cells grown under Pka1-repressed and Pka1-induced conditions compared to all genes present in the WT strain. The enrichment is based on GO terms associated with biological processes

Based on our identification and quantification of 192 proteins in the secretome of *C. neoformans*, we next sought to classify the corresponding genes according to their GO terms of biological process, cellular component, and molecular function. Our goal was to assess whether subsets of genes showed significant over-representation relative to all genes in *C. neoformans*. To perform the enrichment analysis, all unique proteins identified under Pka1-repressed conditions were combined into a single data set as were proteins identified under Pka1-induced conditions. As shown in Fig. 3, the identified secreted proteins under Pka1-repressed conditions were enriched in 15 biological categories, with the most significant enrichment associated with carbohydrate metabolic process, catabolic process, generation of precursor metabolites and energy, organic substance metabolic process, and primary metabolic process. Under Pka1-induced conditions, enrichment was only associated with the five most significantly enriched categories under Pka1-repressed conditions. Classification by cellular components showed the most significant enrichment associated with the cytoplasm under both conditions, which may be an artifact of the classification process or indicative of the location of protein synthesis (see Additional file 6: Figure S1), whereas classification by molecular function showed no enrichment.

**Fig. 4:**
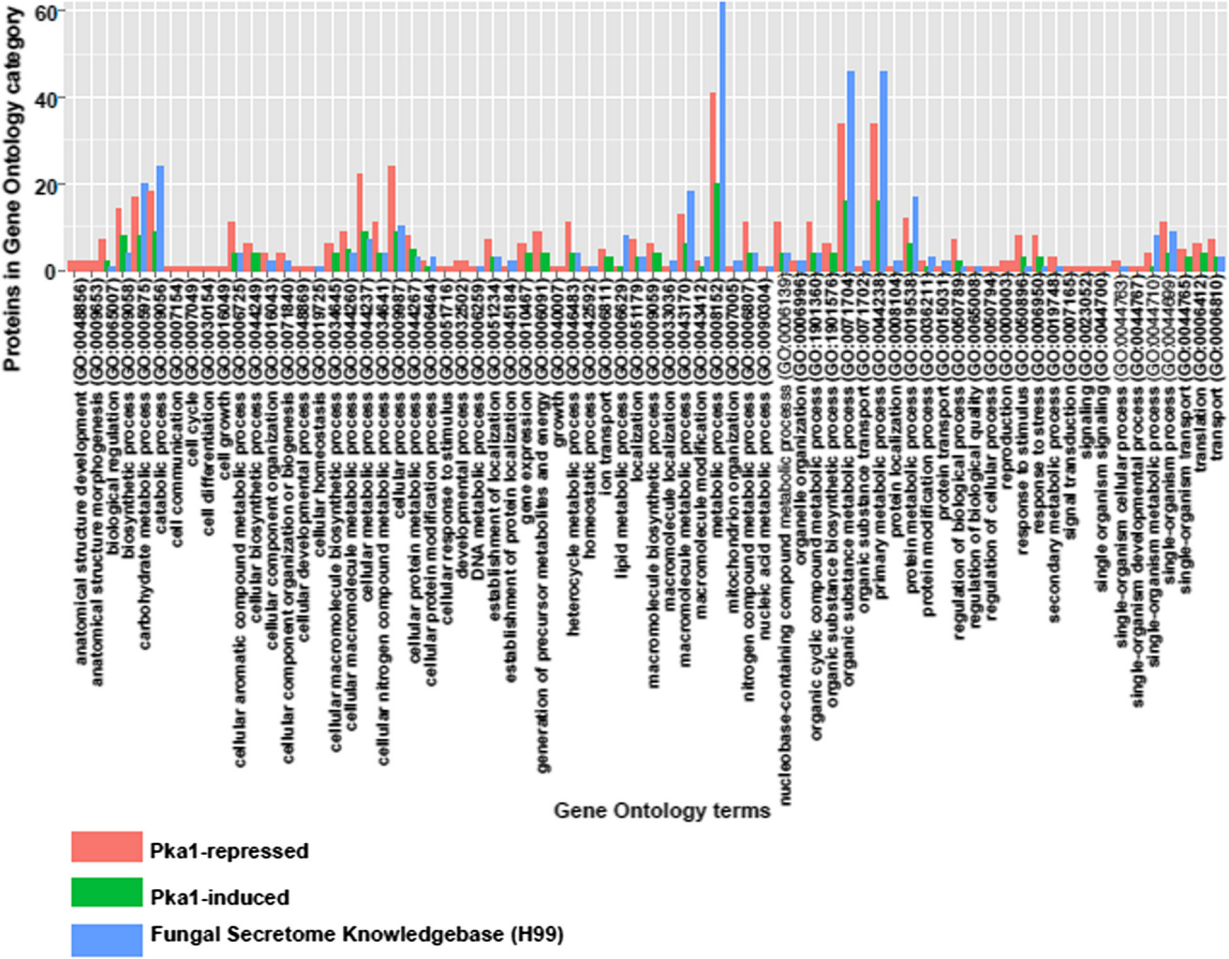
Comparison of GO terms classifications of biological processes from the identified secreted proteins from cells grown under Pka1-repressed and Pka1-induced conditions compared to proteins represented in the Fungal Secretome Knowledgebase

Our gene sets were also compared to all reported secreted proteins in the Fungal Secretome Knowledge Base (FunSecKB) for *C. neoformans* strain JEC21 [31, 32]. The analysis showed no significant enrichment; however, similarities among the identified GO terms were observed (Fig. 4). Forty-seven GO term categories were shared between the FunSecKB and our identified proteins under Pka1-repressed and Pka1-induced conditions; the greatest number of proteins being associated with metabolic processes. Twenty-five categories were represented only in our secretome data, and one category (GO:0009607; response to biotic stimulus) was represented only in the FunSecKB. Upon comparison of GO term categories for cellular components, 16 categories were shared between the FunSecKB and our identified proteins under Pka1-repressed and Pka1-induced conditions; the greatest number of proteins being associated with the cell, cytoplasm, and intracellular categories (see Additional file 7: Figure S2). Upon comparison of GO term categories for molecular function, 17 categories were shared between the FunSecKB and our identified proteins under Pka1-repressed and Pka1-induced conditions; the greatest number of proteins associated with binding as well as enzyme activity (see Additional file 8: Figure S3). Taken together, the enrichment analysis of our secretome data under modulation of Pka1 activity compared to the whole genome showed over-representation of genes associated with a broad spectrum of processes including metabolic and catabolic processing. Although no enrichment was observed between our secretome data and the FunSecKB, a comparison of GO terms between the data sets showed all but one of our identified proteins to be represented in the FunSecKB.

### A bioinformatic analysis of the secretome predicts modes of secretion

We next examined the secreted proteins, under modulation of Pka1 activity, for the presence of predicted signal peptides and GPI anchors. Specifically, we used SignalP 4.1, Signal-3 L, and Phobius for the prediction of protein extracellular location based on the presence or absence of N-terminal signal peptides. The presence of a signal peptide suggests conventional secretion versus potential non-conventional export if a signal peptide is absent. Additionally, we used GPI-SOM to predict the presence or absence of a GPI-anchor on proteins, indicative of plasma membrane association, which may or may not be capable of dissociation and subsequent protein secretion. Of the 61 proteins used for this analysis, 14 had both an N-terminal signal peptide and a GPI-anchor protein, 17 had only an N-terminal signal peptide, one had a GPI-anchor but no N-terminal signal peptide, and 29 proteins did not have an N-terminal signal peptide or a GPI-anchor (Table 4). Taken together, these results suggest that *C. neoformans* may employ a non-conventional secretory pathway for regulation of part of its secretome, including potential protein secretion via vesicle export [24].

**Table 4:** Bioinformatic analysis of identified and quantified proteins in the secretome of *C. neoformans* under Pka1-repressed and Pka1-induced conditions

GO categories^a^	Accession number	Protein name	Signal peptide^b^ (position)	GPI anchor^c^ (position)	Sample preparation
Carbohydrate catabolic process					
	CNAG_02189	α-Amylase	Yes (21/22)	Yes (C-26)	EtOH, TCA/acetone
GTP catabolic process					
	CNAG_06125	Translation elongation factor 1 α	No	No	EtOH, TCA/acetone
	CNAG_06840	Translation elongation factor 2	No	No	TCA/acetone
Carbohydrate metabolic process					
	CNAG_00799	Cellulase	Yes (22/23)	No	TCA/acetone
	CNAG_01239	Chitin deacetylase	Yes (18/19)	Yes (C-28)	EtOH, TCA/acetone
	CNAG_02860	Endo-1,3(4)-β-glucanase	Yes (22/23)	No	EtOH
	CNAG_04245	Chitinase	Yes (21/22)	No	EtOH, TCA/acetone
	CNAG_05799	Chitin deacetylase	Yes (18/19)	Yes (C-28)	EtOH
	CNAG_06291	Deacetylase	Yes (19/20)	No	EtOH, TCA/acetone
	CNAG_06313	Phosphoglucomutase	No	No	TCA/acetone
	CNAG_06501	1,3-β-glucanosyltransferase	Yes (20/21)	Yes (C-6)	EtOH, TCA/acetone
Cellular carbohydrate metabolic process					
	CNAG_03225	Malate dehydrogenase	No	No	EtOH, TCA/acetone
Trehalose metabolic process					
	CNAG_03525	Trehalase	Yes (18/19)	No	TCA/acetone
Glyoxylate metabolic process					
	CNAG_01137	Aconitase	No	No	TCA/acetone
Pentose-phosphate pathway					
	CNAG_01984	Transaldolase	No	No	TCA/acetone
	CNAG_07561	Phosphogluconate dehydrogenase	No	No	EtOH
Protein ubiquitination					
	CNAG_01920	Polyubiquitin	No	No	EtOH
ATP hydrolysis coupled proton transport					
	CNAG_05750	ATPase α subunit	No	No	EtOH, TCA/acetone
	CNAG_05918	F0F1 ATP synthase subunit β	No	No	EtOH
Transmembrane transport					
	CNAG_02974	Voltage-dependent ion-selective channel	No	No	TCA/acetone
	CNAG_06101	Eukaryotic ADP/ATP carrier	No	Yes (C-25)	EtOH, TCA/acetone
Methionine biosynthetic process					
	CNAG_01890	5-methyltetrahydropteroyltriglutamate-homocysteine S-methyltransferase	No	No	EtOH
Dephosphorylation					
	CNAG_02944	Acid phosphatase	Yes (16/17)	No	EtOH, TCA/acetone
Glycolysis					
	CNAG_03072	Phosphopyruvate hydratase	No	No	EtOH, TCA/acetone
	CNAG_06699	Glyceraldehyde-3-phosphate dehydrogenase	No	No	TCA/acetone
Oxidation-reduction process					
	CNAG_01019	Cu/Zn superoxide dismutase	No	No	EtOH
	CNAG_03465	Laccase	Yes (20/21)	No	EtOH, TCA/acetone
Proteolysis					
	CNAG_00581	Endopeptidase	Yes (19/20)	No	TCA/acetone
	CNAG_00919	Carboxypeptidase D	Yes (21/22)	No	EtOH, TCA/acetone
Response to stress					
	CNAG_00334	Heat shock protein	No	No	TCA/acetone
	CNAG_01727	Hsc70-4	No	No	EtOH
	CNAG_01750	Chaperone	No	No	EtOH, TCA/acetone
	CNAG_06150	Heat-shock protein 90	No	No	TCA/acetone
Translation					
	CNAG_04021	60S ribosomal protein L26	No	No	TCA/acetone
	CNAG_04114	40S ribosomal protein S0	No	No	TCA/acetone
	CNAG_06095	Ribosomal protein L13	No	No	EtOH
	CNAG_06605	Ribosomal protein S2	No	No	TCA/acetone
Regulation of transcription					
	CNAG_00483	Actin	No	No	TCA/acetone
Nucleosome assembly					
	CNAG_06746	Histone h2b	No	No	TCA/acetone
Unknown/Unclassified					
	CNAG_00407	Glyoxal oxidase	Yes (16/17)	Yes (C-27)	EtOH, TCA/acetone
	CNAG_00776	Immunoreactive mannoprotein MP88	Yes (22/23)	Yes (C-30)	TCA/acetone
	CNAG_01653	Cytokine-inducing glycoprotein	Yes (19/20)	No	EtOH
	CNAG_02030	Glyoxal oxidase	Yes (21/22)	Yes (C-32)	EtOH, TCA/acetone
	CNAG_02850	Glucan endo-1,3-α-glucosidase agn1	Yes (21/22)	Yes (C-26)	TCA/acetone
	CNAG_02864	Predicted protein	Yes (16/17)	No	TCA/acetone
	CNAG_02943	Cytoplasmic protein	No	No	TCA/acetone
	CNAG_04291	Glycosyl-hydrolase	Yes (17/18)	Yes (C-31)	EtOH
	CNAG_04753	Lactonohydrolase	No	No	EtOH, TCA/acetone
	CNAG_06267	Rds1 protein	Yes (20/21)	Yes	EtOH, TCA/acetone
Hypothetical					
	CNAG_00586	Conserved hypothetical protein	Yes (23/24)	No	EtOH, TCA/acetone
	CNAG_00587	Conserved hypothetical protein	Yes (19/20)	No	TCA/acetone
	CNAG_00588	Conserved hypothetical protein	Yes (16/17)	No	EtOH
	CNAG_01047	Conserved hypothetical protein	Yes (19/20)	No	EtOH, TCA/acetone
	CNAG_01562	Conserved hypothetical protein	Yes (18/19)	No	EtOH
	CNAG_03007	Conserved hypothetical protein	No	No	EtOH
	CNAG_03223	Conserved hypothetical protein	Yes (19/20)	Yes (C-18)	EtOH
	CNAG_03492	Conserved hypothetical protein	Yes (19/20)	No	TCA/acetone
	CNAG_05312	Conserved hypothetical protein	Yes (19/20)	Yes (C-19)	EtOH, TCA/acetone
	CNAG_05595	Conserved hypothetical protein	Yes (18/19)	Yes (C-29)	TCA/acetone
	CNAG_05893	Conserved hypothetical protein	Yes (16/17)	Yes (C-13)	EtOH, TCA/acetone
	CNAG_06109	Conserved hypothetical protein	No	No	EtOH

^a^Proteins were categorized based on GO terms associated with their biological classification

^b^The presence of a signal peptide was determined using SignalP, Phobius, and Signal-3 L

^c^The presence of a signal peptide was determined using GPI-SOM

### Examination of transcription and protein abundance in the context of Pka1 regulation

**Fig. 5:**
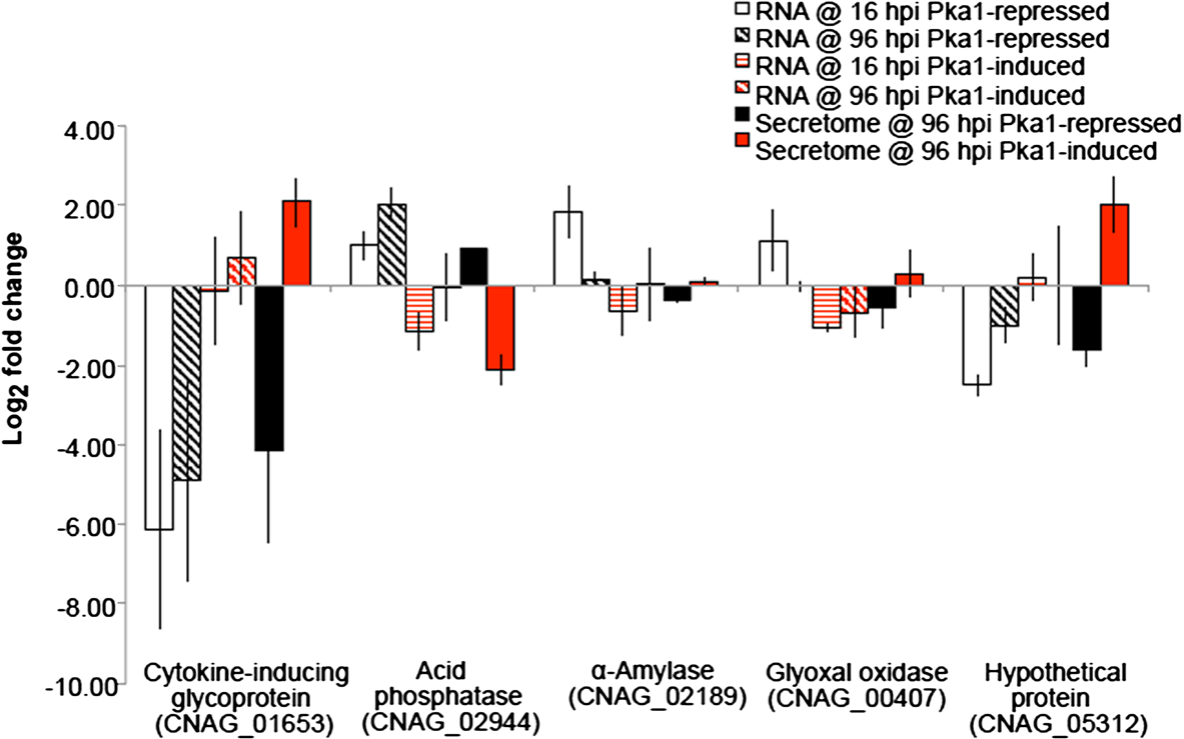
Comparison of RNA expression levels using qRT-PCR to analyze samples from cells at 16 and 96 hpi, versus secreted protein abundance using quantitative proteomics at 96 hpi under Pka1-repressed and Pka1-induced conditions. The samples were evaluated in triplicate, and values are reported as average log_2_ quantification ± standard deviation

Based on our identification and quantification of five secreted proteins regulated by Pka1 in *C. neoformans*, we evaluated whether transcript levels were also influenced by Pka1 regulation and whether there was a correlation with the observed regulation of protein abundance. Specifically, we performed qRT-PCR on RNA collected at 16 and 96 hpi from cells grown in Pka1-repressed and Pka1-induced conditions for the WT and *P*_*GAL7*_*::PKA1* strains, and compared the observed values to our quantitative proteomic results at 96 hpi. Figure 5 summarizes the RNA expression levels at 16 hpi and 96 hpi and protein abundance at 96 hpi for Cig1, the acid phosphatase Aph1, an α-amylase, a glyoxal oxidase, and a novel protein (CNAG_05312). Cig1 and the novel protein both showed down-regulation of their transcripts under Pka1-repressed conditions at 16 and 96 hpi, followed by minimal or slight up-regulation with induced Pka1 activity. α-Amylase and glyoxal oxidase showed an initial peak in transcript levels at 16 hpi, followed by minimal change or a decrease in RNA levels at 96 hpi under Pka1-repressed conditions, and the transcript levels decreased in response to Pka1 induction. Acid phosphatase showed elevated transcript levels upon *PKA1* repression at both time points, compared to a drop in RNA levels at 16 hpi or no change at 96 hpi upon induction of *PKA1*. In general, Pka1 appears to positively regulate the transcript levels of Cig1 and the novel protein (CNAG_05312), and to negatively regulate the transcript levels of the other three proteins. Taken together, our results suggest that although Pka1 activity influences the transcript levels and extracellular abundance of the five proteins, a correlation between transcript and protein levels was not always observed, and this was particularly notable for glyoxal oxidase. The differences may indicate additional levels of potential influence of Pka1 beyond transcriptional regulation, including differences in mRNA versus protein stability, the timing of expression and the regulation of protein export. For example, more detailed studies will be needed to examine the timing of intracellular and extracellular accumulation of the glyoxal oxidase protein relative to transcription of the gene.

### Detection of secreted Pka1-regulated proteins using Multiple Reaction Monitoring

Based on our identification of five Pka1-regulated proteins, including two with roles in virulence, we hypothesized that these proteins would be secreted during infection and that they might be potentially useful biomarkers of cryptococcosis. To test this idea, we used Multiple Reaction Monitoring (MRM), a powerful and targeted proteomics approach for the relative quantitative measurement of target proteins. In the presence of an internal standard, a stable isotope-labeled peptide, the amount of natural protein can be measured by comparing the signals to the labeled species. The isotopically labeled, proteotypic peptides terminate with C-terminal heavy arginine or lysine (C-term Arg U-^13^C_6_;U-^15^N_4_ or Lys U-^13^C_6_;U-^15^N_2_). In principle, the stable isotopes have the same physiochemical properties as the natural peptides and only differ by mass resulting in co-elution of the peptides. However, studies have suggested that in the presence of complex biological samples, such as blood or serum, the retention times between the peptides can shift, impacting the co-elution patterns [33]. We specifically applied MRM to detect Cig1, Aph1, glyoxal oxidase, α-amylase, and the novel protein (CNAG_05312) in samples from a macrophage-like cell line and from infected mice.

**Fig. 6:**
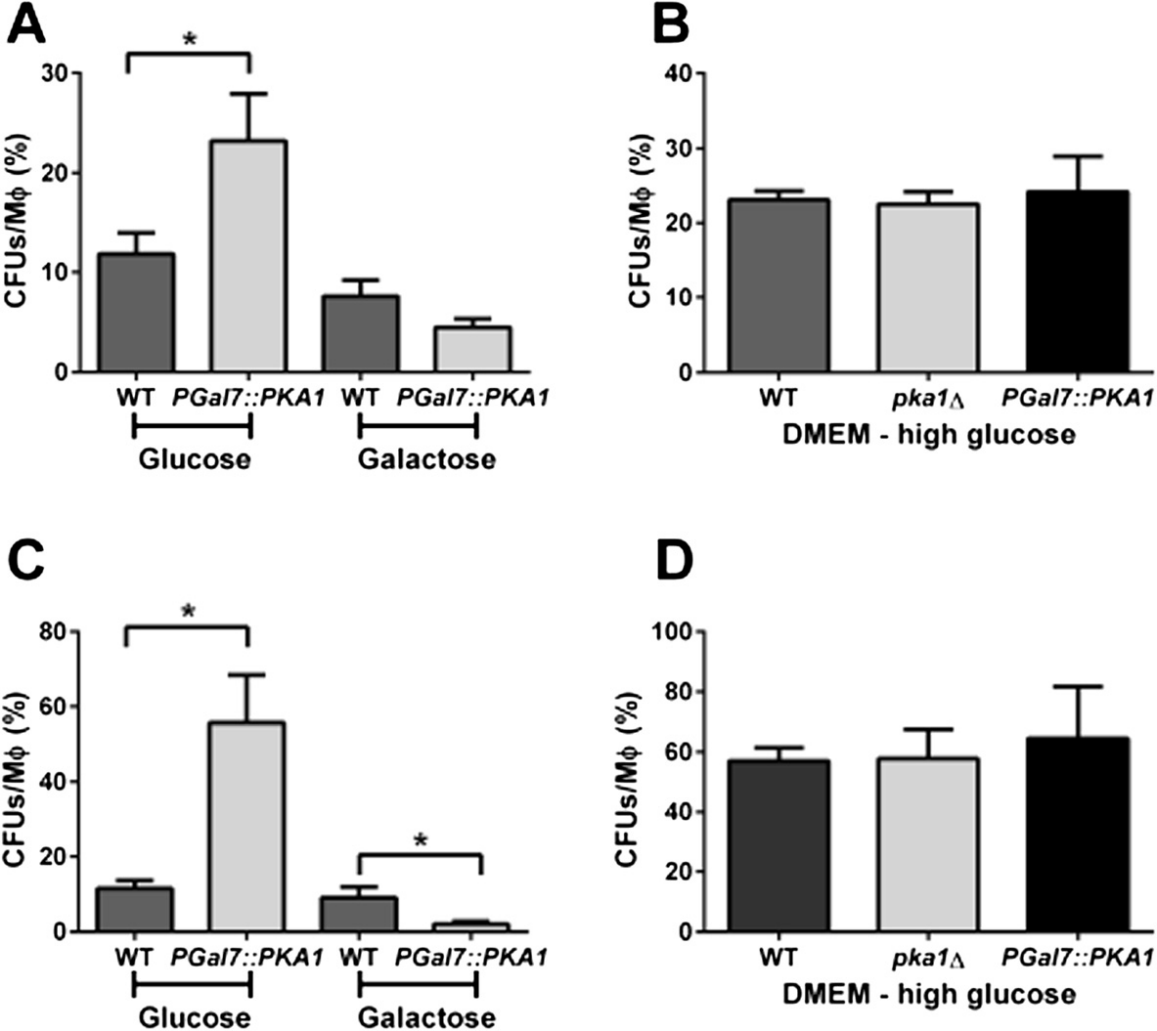
Interactions of WT and Pka1-regulated strains with J774A.1 murine macrophages. **a** Intracellular rate at 2 hpi of WT and *P*_*GAL7*_ *::PKA1* strains grown under Pka1-repression (glucose) and Pka1-induction (galactose). **b** As a control, the colony forming units (CFUs) per macrophage grown in standard DMEM medium (containing 0.45 % glucose) are presented. **c** Rate of intracellular fungal cell per macrophage at 24 hpi of WT and *P*_*GAL7*_ *::PKA1* strains grown under Pka1-repression (glucose) and Pka1-induction (galactose). **d** The CFUs per macrophage grown in standard DMEM medium (containing 0.45 % glucose) are presented as a control. The experiments were performed in triplicate; the average percent of survival was reported ± standard error of the mean. For statistical analysis, an unpaired *t-test* with Welch’s correction (*p-value* < 0.05) was performed between conditions (* denotes significant difference). The samples at 24 hpi were employed for the analysis of protein abundance shown in Fig. 7

The samples from the J774A.1 macrophage-like cell line came from cells inoculated with WT and *P*_*GAL7*_*::PKA1* strains under Pka1-repressed (DMEM medium supplemented with glucose) and Pka1-induced (DMEM medium supplemented with galactose) conditions. Intracellular uptake at 2 hpi showed a significant difference in the number of colony forming units (CFUs) per macrophage between the WT and *P*_*GAL7*_*::PKA1* strains under Pka1-repressed conditions, but not under induced conditions (Fig. 6a). This difference is most likely due to the absence of the capsule for the Pka1-repressed cells, a phenotype that enhances phagocytosis. By 24 hpi, rates of intracellular fungal cells per macrophage were significantly different for WT and *P*_*GAL7*_*::PKA1* strains under both conditions (Fig. 6c). Specifically, intracellular rates of infection at 24 hpi in repressed conditions were 11.49 ± 2.11 % for the WT and 55.67 ± 12.76 % for *P*_*GAL7*_*::PKA1* strains. However, intracellular rates under induced conditions were 9.06 ± 2.91 % for WT and 1.97 ± 0.82 % for *P*_*GAL7*_*::PKA1* strains. Importantly, intracellular uptake rates showed no differences between WT, *P*_*GAL7*_*::PKA1*, and the *pka1Δ* strains under controlled growth conditions (DMEM – high glucose (0.45 %)) at 2 and 24 hpi (Fig. 6b, d). These results indicate that modulation of *PKA1* expression influences the intracellular survival of cryptococcal cells.

**Fig. 7:**
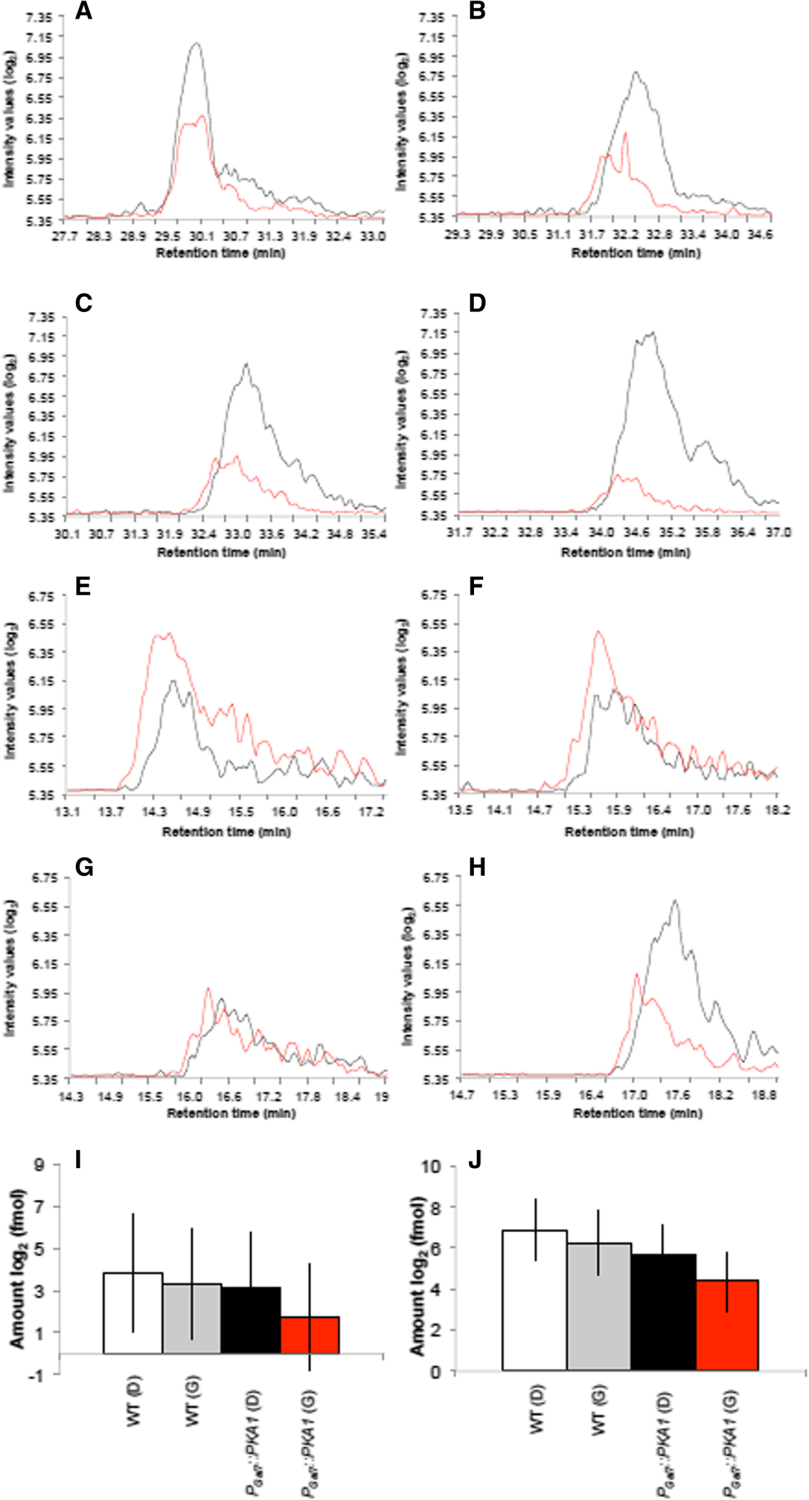
Detection of Pka1-regulated proteins in lysates of macrophage-like cells containing *C. neoformans*. Chromatographic representation of the most abundant peptide and its transition for α-amylase (CNAG_02189) identified from isotopically-labeled peptide or natural peptide for each of the following samples: **a** macrophage lysate challenged with WT cells grown in 0.2 % glucose; (**b**) macrophage lysate challenged with WT cells grown in 0.2 % galactose; (**c**) macrophage lysate challenged with *P*_*GAL7*_ *::PKA1* cells grown in 0.2 % glucose; (**d**) macrophage lysate challenged with *P*_*GAL7*_ *::PKA1* cells grown in galactose 0.2 %. Chromatographic representation of the most abundant peptide and its transition for glyoxal oxidase (CNAG_00407) identified from isotopically-labeled peptide or natural peptide for each of the following samples: (**e**) macrophage lysate challenged with WT cells grown in 0.2 % glucose; (**f**) macrophage lysate challenged with WT cells grown in 0.2 % galactose; (**g**) macrophage lysate challenged with *P*_*GAL7*_ *::PKA1* cells grown in 0.2 % glucose; (**h**) macrophage lysate challenged with *P*_*GAL7*_ *::PKA1* cells grown in 0.2 % galactose. Black indicates isotopically-labeled peptide; red indicates natural peptide. **i** Quantification of α-amylase identified in the macrophage lysates was based on the area under the curve for the isotopically-labeled peptide versus the natural peptide in WT and *P*_*GAL7*_ *::PKA1* strains under Pka1-repressed (**d**) and Pka1-induced (**g**) conditions. The avergae (± S.D.) amount of peptide present in the sample is reported. **j** Quantification of glyoxal oxidase identified in the macrophage lysates was based on the area under the curve for the isotopically-labeled peptide versus the natural peptide in WT and *P*_*GAL7*_ *::PKA1* strains under Pka1-repressed (D) and Pka1-induced (G) conditions. The avergae (± S.D.) amount of peptide present in the sample is reported. Five micrograms of total protein were used for the assays and all assays were performed in triplicate

MRM on macrophage lysates infected with fungal cells at 24 hpi identified the Pka1-regulated and secreted proteins α-amylase and glyoxal oxidase in both induced and repressed conditions. Figure 7 shows representative chromatographic co-elution patterns of the isotopically-labeled and natural peptides, which allowed for relative quantification of peptides in the replicates of the experiment. For both enzymes, the highest amount of protein was detected in the WT strain in DMEM medium under Pka1-repressed conditions, whereas the *P*_*GAL7*_*::PKA1* strain under Pka1-induction showed the lowest amount of secreted protein. This observation may be associated with reduced intracellular rates of the *P*_*GAL7*_*::PKA1* strain due to the presence of an enlarged capsule. Overall, we were able to detect 29.8 ± 37.0 fmol of α-amylase and 149.1 ± 130.0 fmol of glyoxal oxidase in 5 μg of total protein from the macrophage lysate following the uptake of *P*_*Gal7*_*::PKA1* under Pka1-induced conditions at 24 hpi.

**Fig. 8:**
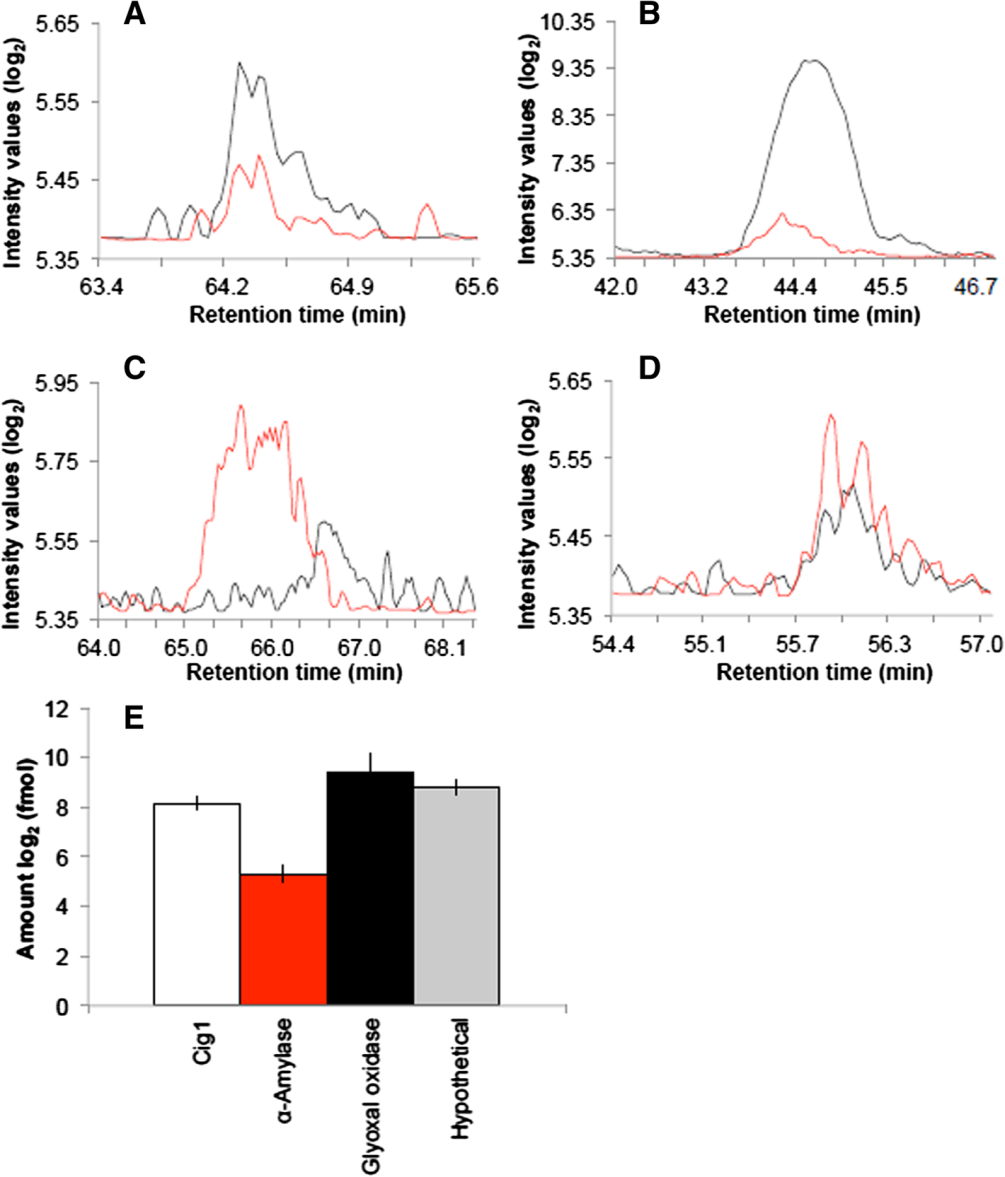
Detection of Pka1-regulated proteins in mouse bronchoalveolar lavage samples. Chromatographic representation of the most abundant peptide and its transition identified from isotopically-labaled peptide or natural peptide for each of the following proteins: (**a**) Cig1 (CNAG_01653), (**b**) α-Amylase (CNAG_02189), (**c**) Glyoxal oxidase (CNAG_00407), (**d**) Hypothetical protein (CNAG_05312). Black indicates isotopically-labeled peptide; red indicates natural peptide. **e** Quantification of proteins identified in the mouse BAL samples based the area under the curve for the isotopically-labeled peptide versus the natural peptide, for Cig1, α-amylase, glyoxal oxidase, and hypothetical (CNAG_05312) proteins. The avergae (± S.D.) amount of peptide present in the sampleis reported. Five micrograms of total protein were used for the assays and all assays were performed in triplicate

**Fig. 9:**
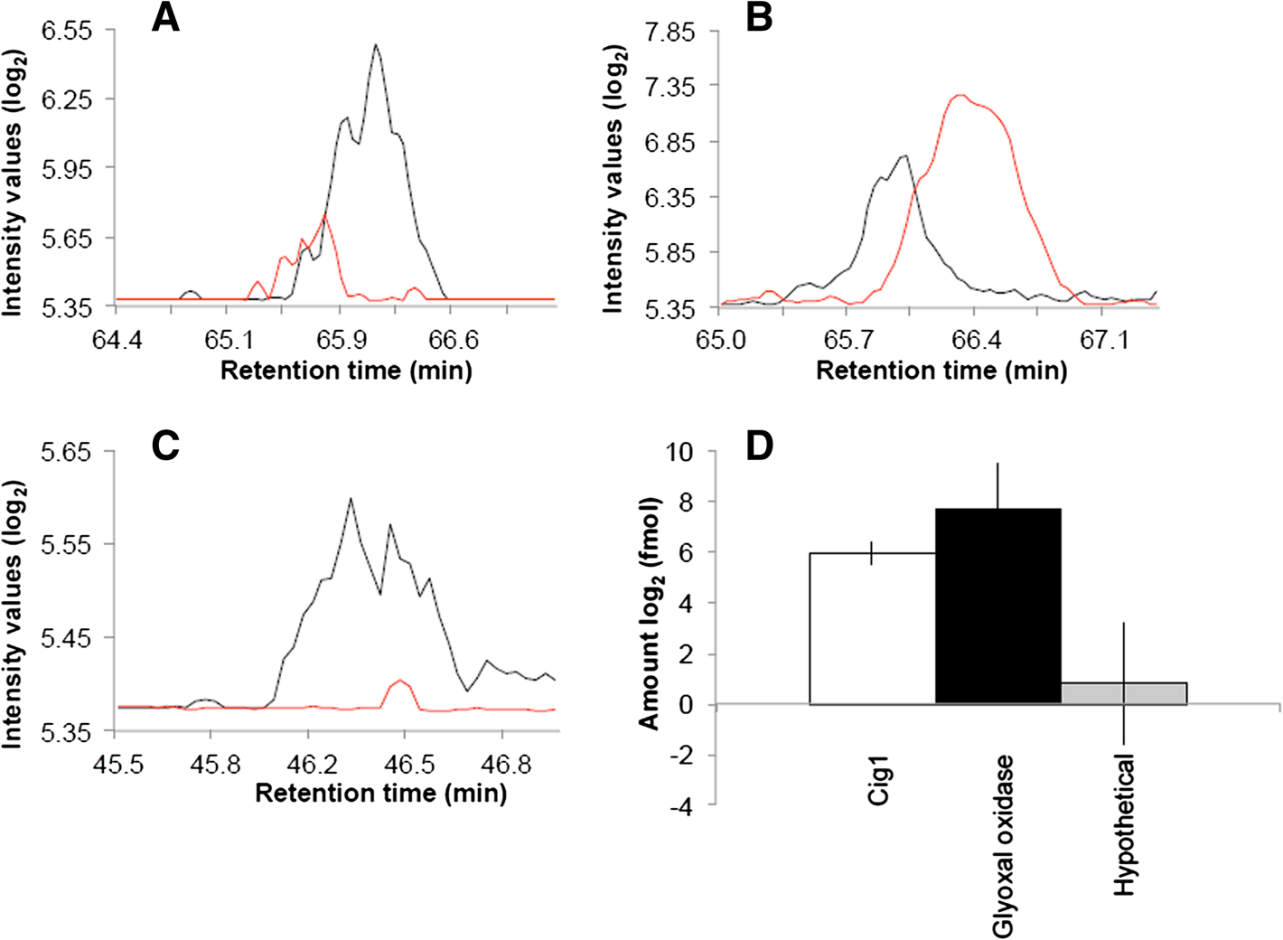
Detection of Pka1-regulated proteins in mouse blood samples. Chromatographic representation of the most abundant peptide and its transition identified from isotopically-labaled peptide or natural peptide for each of the following proteins: (**a**) Cig1 (CNAG_01653), (**b**) Glyoxal oxidase (CNAG_004070), (**c**) Hypothetical protein (CNAG_05312). Black indicates isotopically-labeled peptide; red indicates natural peptide. **d** Quantification of proteins identified in the mouse blood samples based the area under the curve for the isotopically-labeled peptide versus the natural peptide, for Cig1, glyoxal oxidase, and hypothetical (CNAG_05312) proteins. The average (± S.D.) amount of peptide present in the sample is reported. Five micrograms of total protein were used for the assays and all assays were performed in triplicate

The samples from infected mice included BAL and blood from animals inoculated with the WT strain. Three mice were selected for each type of *in vivo* analysis based on previous studies of cryptococcosis [34–37]. Representative chromatograms of isotopically-labeled and natural peptides detected in mouse BAL are presented in Fig. 8. The MRM analysis identified Cig1, α-amylase, glyoxal oxidase, and the novel protein (CNAG_05312) in BAL following infection with WT cells. In 5 μg of total protein, glyoxal oxidase was the most abundant protein with detection at 779.5 ± 436.1 fmol, followed by the novel protein (CNAG_05312) at 451.0 ± 90.5 fmol, Cig1 at 291.3 ± 54.5 fmol, and α-amylase with the lowest abundance at 40.1 ± 9.4 fmol. Lastly, we were able to detect Cig1, glyoxal oxidase, and the novel protein (CNAG_05312) in blood. Representative chromatograms of the isotopically-labeled and natural peptides detected in mouse blood are presented in Fig. 9. Again, glyoxal oxidase was the most abundant protein detected at 319.4 ± 272.7 fmol, followed by Cig1 at 62.0 ± 17.4 fmol, and the novel protein (CNAG_05312) at 3.1 ± 3.8 fmol in 5 μg of total protein. Aph1 levels were below the limit of detection in all samples. Taken together, our targeted proteomics approach identified and quantified the Pka1-regulated secreted proteins as potential biomarkers following host challenge with cryptococcal cells.

## Discussion

The secretion of extracellular enzymes and virulence-associated factors is important for the proliferation and survival of pathogens in the host environment. For the pathogenic yeast *C. neoformans,* virulence depends to a large extent on the export of polysaccharide to form a capsule, as well as targeted delivery of laccase to the cell wall for deposition of melanin, and secretion of extracellular enzymes [23, 24, 28, 38]. The cyclic-AMP/Protein Kinase A signal transduction pathway plays a key role in regulating these processes but the underlying mechanisms remain to be understood in detail [16, 17]. We therefore used a *P*_*GAL7*_*::PKA1* strain under Pka1-repressed and Pka1-induced conditions in this study to investigate the influence of Pka1 on the secretome of *C. neoformans*. Quantitative proteomics allowed us to identify 61 different proteins in the secretome including a subset of five whose abundance was regulated by Pka1. These five proteins include a cytokine-inducing glycoprotein (Cig1), an α-amylase, a glyoxal oxidase, an acid phosphatase (Aph1), and a novel protein (CNAG_05312). We also observed a change in the secretome profile upon induction of *PKA1* expression thus establishing a view of the impact of PKA activity on the extracellular protein composition. In general, this analysis highlighted the enrichment of Pka1-regulated biological processes in the secretome, revealed potential targets for conventional and non-conventional modes of secretion, and provided candidate biomarkers for investigating cryptococcosis.

### Modulation of *PKA1* expression leads to a change in the secretome

Our analysis revealed a change in the abundance of secreted *C. neoformans* proteins associated with glycolysis, translational regulation, nucleosome assembly, and stress response over a time course from 16 to 120 h. We speculate that some of these proteins may result from packaging in vesicles known to transit through the cell wall and accumulate in the extracellular environment [24, 39]. In this case, modulation of PKA activity may indirectly influence the proteome of vesicles as a reflection of an impact on the intracellular proteome. This idea is supported by our observed influence of *PKA1* modulation on the abundance of the translation machinery because ribosomal proteins, in particular, are abundant in extracellular vesicles [39]. It is also well known that PKA influences the transcription of ribosomal protein genes in other organisms and this influence is conserved in *C. neoformans* [22, 40]. Our analysis of the intracellular proteome also revealed suppression of ribosomal cellular protein abundance upon induction of Pka1 (Geddes et al., unpublished data). We also observed a connection between Pka1 activation and the abundance of glycolytic proteins. This is interesting in light of previous reports demonstrating the importance of glycolysis for virulence and the persistence of *C. neoformans* in the cerebral spinal fluid [41]. These findings are consistent with a previous analysis of the transcriptome, which showed that Pka1 influences the levels of transcript for genes involved in glycolysis [22]. Furthermore, the observed influence of Pka1 induction on the secretion of proteins associated with stress response is consistent with observed Pka1 regulation at the transcriptional level. In this context, we identified a heat shock protein 70 (Hsc70-4), which is associated with the response to stress and which was previously localized to the cell surface of *C. neoformans* [42]. The observed connection between the stress response and Pka1 induction may indicate coordination for facilitation of fungal survival and proliferation during colonization of vertebrate hosts.

### Pka1 regulation of mannoproteins and cell wall functions: connections with Rim101

The influence of PKA on the abundance of the mannoprotein Cig1 is of particular interest because we previously showed that its transcript is one of the most abundant in cells grown in low iron medium [43]. In addition, the protein is important for iron acquisition from heme and virulence in *C. neoformans* [44]. We found that the extracellular abundance of Cig1 increased upon induction of Pka1 and that transcript levels and protein abundance were well correlated. *CIG1* is positively regulated by the pH-responsive transcription factor Rim101, which in turn is activated by the cAMP/PKA pathway [45]. Therefore, the regulation of *CIG1* mRNA and Cig1 protein levels observed upon induction of Pka1 likely reflect regulation by Rim101. This finding is consistent with recent discoveries that Rim101 controls cell wall composition and capsule attachment via an influence on the expression of cell wall biosynthetic genes [46, 47].

In general, a number of proteins associated with cell wall synthesis and integrity, pathogenesis and the immune response were prominent in the secretome of *C. neoformans* upon modulation of *PKA1* expression. These proteins included an endo-1,3(4)-β glucanase and a 1,3-β-glucanosyltransferase, both of which have been previously identified in studies of the extracellular proteomes of *C. neoformans* and other fungal pathogens such as *Histoplasma capsulatum* [28, 48–51]. Endo-1,3(4)-β glucanase is located in the surface layers of the cell wall or in the capsule and has roles in metabolism, autolysis, and cell separation [50, 52]. The 1,3-β-glucanosyltransferase is described as a glycolipid protein anchored to the cell membrane in yeasts and may have a role in virulence [53]. Our proteomic analysis also identified chitin deacetylases associated with the formation of chitin and cell wall integrity, and the enzyme laccase, which is responsible for melanin deposition in the cell wall and influences cryptococcal virulence [28, 51, 54–57]. These findings are consistent with our previous transcriptomic analysis, which revealed an influence of PKA on the expression of cell wall associated genes [22].

We also identified a novel protein (CNAG_05312) with a pattern of mRNA and protein regulation by Pka1 activity that was quite similar to that of Cig1. This novel protein contains a predicted carbohydrate-binding domain and was annotated as a macrophage-activating glycoprotein (reminiscent of the cytokine-inducing glycoprotein designation of Cig1). These observations suggest that further investigation is warranted for this protein in the context of iron acquisition and virulence. This idea is reinforced by the finding that Rim101 also positively regulates expression of the CNAG_05312 gene [45]. Interestingly, the CNAG_05312 gene is also regulated at the transcript level by the transcription factor Gat201 that, like Pka1, influences capsule size, virulence, and uptake by macrophages [58, 59]. Considering these similar phenotypes, it is possible that Gat201 and Pka1/Rim101 both regulate the expression of the CNAG_05312 protein and subsequently influence the activation of macrophages during infection. Overall, our investigation of the secretome reinforced connections between modulation of Pka1 activity, Rim101 and cell wall integrity, and it revealed an impact of PKA on the extracellular abundance of proteins with known (Cig1) and potential (the novel CNAG_05312 protein) influences on virulence.

### Pka1 influences the secretion of α-amylase and glyoxal oxidase enzymes

Pka1 also positively regulated the abundance in the secretome of an α-amylase and a glyoxal oxidase which were previously identified in the extracellular proteome of *C. neoformans* [28, 51]. Amylases are associated with carbohydrate metabolism, particularly starch degradation for energy production [60]. In *C. neoformans*, the secretion of amylases in the PKA-regulated strains was reported previously and we were able to measure and confirm α-amylase activity in the extracellular medium [29]. Glyoxal oxidases are extracellular H_2_O_2_-producing enzymes associated with cellulose metabolism [61]. There is evidence that glyoxal oxidase activity is involved in filamentous growth and pathogenicity of *Ustilago maydis*, as well as fertility in *Cryptococcus gattii* [61, 62]. A similar pattern in response to *PKA1* expression was observed upon comparison of the transcript and protein levels for both the α-amylase and the glyoxal oxidase. A direct correlation between transcript levels and protein abundance was not as evident as for Cig1. This could potentially be due to post-transcriptional regulation, differences in mRNA and protein half-lives and issues with timing [63]. It is also possible that PKA may regulate additional processes to influence extracellular protein abundance, such as the activity of the secretory pathway. Overall, the secretome data revealed a new connection between PKA regulation and the α-amylase and glyoxal oxidase enzymes, and this discovery indicates that further analysis of their potential roles in virulence is warranted.

### Pka1 influences the secretion of the virulence-associated acid phosphatase, Aph1

The extracellular abundance of the acid phosphatase Aph1 and its transcript levels were negatively regulated by induction of *PKA1* expression thus revealing an opposite pattern of regulation compared with the other four genes. Phosphatases have been predicted to have roles in cell wall biosynthesis, cell signaling, phosphate scavenging, and in adhesion of *C. neoformans* to epithelial cells [24, 28, 64–67]. The *APH1* gene was recently characterized and its expression was found to be induced by phosphate limitation; the Aph1 protein was also the major conventionally secreted acid phosphatase in *C. neoformans* [28]*.* Aph1 was also shown to hydrolyze a variety of substrates to potentially scavenge phosphate from the environment, and an *aph1* deletion mutant had a slight virulence defect in both *Galleria mellonella* and mouse models of cryptococcosis. The latter phenotype is consistent with our recent study showing that a high affinity phosphate uptake system is required for growth on low-phosphate medium, for formation of the virulence factors melanin and capsule, for survival in macrophages, and for virulence in mice [68]. This study also revealed that defects in PKA influence the growth of *C. neoformans* on phosphate-limited medium. Our discovery of PKA regulation of Aph1 abundance in the secretome therefore further reinforces a connection between phosphate acquisition and PKA regulation associated with virulence.

### PKA regulation and the intersection of secretome studies in *C. neoformans*

Our profiling of the secretome upon modulation of Pka1 activity confirmed the presence of previously identified extracellular and vesicular proteins, including those associated with virulence and fungal survival within the host, as well as novel secreted proteins. We identified the classically secreted *C. neoformans* protein, laccase, associated with fungal virulence, but other proteins such as urease and phospholipase B were not identified in our study. Their absence could be attributed to growth conditions, precipitation methods, supernatant collection times, and relative abundance in the secretome. A recent proteome study that removed free capsular polysaccharide from the extracellular environment identified 105 secreted proteins and a direct comparison with our study showed an overlap of 52 % [28]. Previous investigation of the proteins in extracellular vesicles of *C. neoformans* also showed an overlap of nearly 56 % with proteins identified in our study [24, 39]. This overlap is primarily associated with proteins not typically expected in the secretome. For example, ATP subunits/carriers, translation elongation factor, actin, and multiple ribosomal proteins were identified and their presence was attributed to packaging in extracellular vesicles, and not necessarily due to direct secretion. In the absence of an N-terminal signal peptide, proteins may be exported via non-conventional secretion. This may include the use of membrane-bound, extracellular vesicles capable of traversing the cell wall, the possible fusion of multi-vesicular bodies with the plasma membrane, or the capture of cytosolic material to form vesicles (blebbing), as discussed above [23, 24, 69–72]. Taken together, our profile of secreted proteins in *C. neoformans* is in agreement with previous secretome studies. However, our ability to modulate Pka1 activity provides an opportunity to identify novel proteins in the extracellular environment as well as identify proteins specifically regulated by Pka1. This approach led to the unique identification of the novel secreted protein (CNAG_05312) that was specifically associated with modulation of Pka1 activity and not found in other proteomic studies.

### Detection of potential biomarkers during cryptococcal infection

Biomarkers are indicators of normal or pathogenic processes as well as the efficacy of therapy [73]. In this regard, targeted detection of secreted cryptococcal proteins provides an opportunity to identify potential biomarkers for early diagnosis of infection and to monitor antifungal therapy. Early and rapid diagnosis remains limited for systemic fungal infections, such as those caused by *Candida* and *Aspergillus* species, as well as *C. neoformans* and *C. gattii* [74]. Biomarkers of infection by specific fungal species would therefore be valuable for identification and for precise measurements of fungal burden. A recent study using the presence of the cell wall component galactomannan in BAL as a diagnostic tool for invasive fungal disease highlights an opportunity for biomarker discovery in fungal pathogens [75]. Additionally, the use of targeted proteomics (and MRM in particular) is a novel approach to study the secretion of virulence factors in *C. neoformans,* particularly in the context of signaling functions like PKA that sense conditions relevant to the host environment.

The secreted proteins that we identified to be regulated in abundance by Pka1 provide an opportunity to develop diagnostic biomarkers that are also informative about signaling via the cAMP/PKA pathway *in vitro* and during infection. For example, Cig1 is an important candidate biomarker given its abundance in iron-starved cells and its role in virulence through iron acquisition and uptake. Our ability to detect Cig1 in the blood and BAL fluid of infected animals confirms its expression and establishes the protein as a potential biomarker. These findings may also indicate a role for Cig1 in iron uptake in these environments although, interestingly, we did not detect Cig1 in macrophage lysates. Based on our observed differences in intracellular replication, Pka1 seems to impact the intracellular environment of macrophages. In this regard, we did detect the glyoxal oxidase and α-amylase proteins by MRM in macrophages containing cryptococcal cells. Expression of these proteins has not previously been reported during interactions with macrophages, although the production of H_2_O_2_ and induction of oxidative stress via glyoxal oxidase could potentially influence intracellular survival. It is known that oxidative stress induces autophagy in macrophages and can impair phagocytic activity [76, 77]. Additionally, loss of an α-amylase in *H. capsulatum* attenuated the ability of the fungus to kill macrophages and to colonize murine lungs [78]. This influence appeared to be related to the ability to produce α-(1,3)-glucan. The regulation of glyoxal oxidase and α-amylase by Pka1 activity and their detection in macrophage lysates suggests that it would be interesting to examine the roles of these enzymes in intracellular survival and virulence. Our approach with MRM is also informative about tissue specific expression of fungal proteins during disease. In addition to the examples described above, we found that colonization of murine lungs resulted in secretion of α-amylase, glyoxal oxidase and the novel protein from gene CNAG_05312. The novel protein was also found in blood and, given its similar regulation with Cig1 these results suggest future studies on the role of this protein in iron acquisition and virulence.

## Conclusion

In this study we characterized the overall impact of *PKA1* modulation on the secretome and discovered five proteins regulated by Pka1. The identified proteins had known roles associated with cell wall functions, fungal survival within the host, and virulence. Our identification of a novel protein with potential roles in iron uptake and virulence also suggested a previously unknown connection between Pka1 and Gat201. We were also able to detect Pka1-regulated secreted proteins in biological samples as potential biomarkers, providing a new opportunity for diagnosing fungal infection and monitoring disease progression.

### Methods

#### Fungal strains and culture conditions

The *C. neoformans var. grubii* wild-type strain H99 (WT) and the *P*_*GAL7*_*::PKA1* strain with galactose-inducible/glucose repressible expression of *PKA1* were used for this study [16, 29]. The strains were maintained on yeast extract peptone dextrose (YPD) medium (1 % yeast extract, 2 % peptone, 2 % dextrose, and 2 % agar). For studies involving regulation of *PKA1*, cells of the WT and regulated strains were pre-grown overnight with agitation at 30 °C in YPD broth, transferred to yeast nitrogen base medium with amino acids (YNB, Sigma-Aldrich) and incubated overnight with agitation at 30 °C. Cell counts were performed and 5 x 10^7^ cells/ml were transferred to Minimal Medium (MM) (29.4 mM KH_2_PO_4_, 10 mM MgSO_4_ • 7H_2_O, 13 mM glycine, 3 μM thiamine, 0.27 % carbon source) containing either glucose (MM + D) or galactose (MM + G). For end-point studies, cells were incubated with agitation at 30 °C in MM + D or MM + G for 96 h; for time-course studies, cells were incubated with agitation at 30 °C in MM + D or MM + G for 16, 48, 72, and 120 h. Time points were selected based on previous studies on the timing of protein secretion as well as the analysis of proteins in extracellular vesicles of *C. neoformans*, which used samples collected at 48 and 72 h of growth [23, 24, 64]. Samples were collected in triplicate for analysis.

### Protein quantification, precipitation and in-solution digestion

To collect supernatant samples, cells were removed by centrifugation at 3,500 rpm for 15 min at 4 °C and the culture medium was transferred to new tubes; this step was repeated four times until all cell debris had been removed. Supernatant samples were kept on ice and total protein concentration was measured by a BCA-Protein-assay (Pierce). Ultrapure bovine serum albumin was used as a calibration standard. In addition to using two approaches for protein precipitation as described below, we also used a combination of sample collection time points (time-point and end-point analyses) to maximize protein detection and obtain a comprehensive view of the secretome. The first approach involved a time-course study in which a stringent trichloroacetic acid (TCA)/acetone precipitation was performed [79]. In brief, an aliquot of culture supernatant (50 μg total protein) was mixed with five volumes of ice-cold TCA/acetone (20 %/80 % w/v) and incubated overnight at −20 °C. Precipitated proteins were collected by centrifugation at 10,000 rpm for 20 min at 4 °C. The pellet was washed four times with ice-cold acetone, air-dried and stored at −20 °C. The second approach, which was less stringent than the TCA/acetone method, was used for the end-point studies and involved ethanol (EtOH)/acetate precipitation [80]. In brief, an aliquot of culture supernatant (50 μg total protein) was diluted with 4 volumes of absolute EtOH, 2.5 M NaCH_3_COO was used to bring the solution to 50 mM NaCH_3_COO, pH 5.0 and 20 μg of glycogen was added to the sample. Samples were vortexed and incubated at room temperature for 2 h with periodic agitation. Precipitated proteins were collected by centrifugation at 15,000 rpm for 10 min at 4 °C. The pellet was washed twice with EtOH, then air-dried and stored at −20 °C. All supernatant samples were subjected to in-solution digestion using ACS grade chemicals or HPLC grade solvents (Thermo Scientific and Sigma-Aldrich) [81]. In brief, the precipitated protein pellet was solubilized in digestion buffer (1 % sodium deoxycholate, 50 mM NH_4_HCO_3_), incubated at 99 °C for 5 min with agitation, followed by reduction (2 mM of dithiothreitol (DTT) for 25 min at 56 °C), alkylation (4 mM of iodoacetamide (IAA) for 30 min at room temperature in the dark), and trypsinization (0.5 μg/μl of sequencing grade modified trypsin (Promega)) overnight at 37 °C. Based on our results, the TCA/acetone precipitation method appeared to be more stringent, perhaps due to more extensive washing in the protocol.

### Peptide chemical labeling and purification

Digested peptides from supernatants were desalted, concentrated, and filtered on C18 STop And Go Extraction (STAGE) tips [82]. Reductive dimethylation using formaldehyde isotopologues was performed to differentially label peptides from the different experimental conditions. Light formaldehyde (CH_2_O) and medium formaldehyde (CD_2_O) (Cambridge Isotope Laboratories, Andover, MA) were combined with cyanoborohydride (NaBH_3_CN, Sigma-Aldrich) to give a 4 Da difference for labeled peptides [83]. Samples from the WT strain were routinely labeled with light formaldehyde, and *P*_*GAL7*_*::PKA1* samples were labeled with medium formaldehyde. Briefly, eluted and dried STAGE-tip peptides were resuspended in 100 mM triethylammonium bicarbonate, and incubated in 200 mM formaldehyde and 20 mM sodium cyanoborohydride for 90 min in the dark. After labeling, 125 mM NH_4_Cl was added and incubated for 10 min to react with excess formaldehyde, followed by the addition of acetic acid to a pH < 2.5 to degrade sodium cyanoborohydride. For each comparison, equal amounts of labeled peptides were mixed and desalted on C18 STAGE tips.

### Protein identification by liquid chromatography-tandem mass spectrometry (LC-MS/MS) and mass spectrometry data analysis

Purified peptides were analyzed using a linear-trapping quadrupole - Orbitrap mass spectrometer (LTQ-Orbitrap Velos; Thermo Fisher Scientific) on-line coupled to an Agilent 1290 Series HPLC using a nanospray ionization source (Thermo Fisher Scientific). This includes a 2-cm-long, 100-μm-inner diameter fused silica trap column, 50-μm-inner diameter fused silica fritted analytical column and a 20-μm-inner diameter fused silica gold coated spray tip (6-μm-diameter opening, pulled on a P-2000 laser puller from Sutter Instruments, coated on Leica EM SCD005 Super Cool Sputtering Device). The trap column was packed with 5 μm-diameter Aqua C-18 beads (Phenomenex, www.phenomenex.com) while the analytical column was packed with 3.0 μm-diameter Reprosil-Pur C-18-AQ beads (Dr. Maisch, www.Dr-Maisch.com). Buffer A consisted of 0.5 % aqueous acetic acid, and buffer B consisted of 0.5 % acetic acid and 80 % acetonitrile in water. Samples were resuspended in buffer A and loaded with the same buffer. Standard 90 min gradients were run from 10 % B to 32 % B over 51 min, then from 32 % B to 40 % B in the next 5 min, then increased to 100 % B over a 2 min period, held at 100 % B for 2.5 min, and then dropped to 0 % B for another 20 min to recondition the column. The HPLC system included Agilent 1290 series Pump and Autosampler with Thermostat; temperature was set at 6 °C. The sample was loaded on the trap column at 5 μl/min and the analysis was performed at 0.1 μl/min. The LTQ-Orbitrap was set to acquire a full-range scan at 60,000 resolution from 350 to 1600 Th in the Orbitrap to simultaneously fragment the top ten peptide ions by CID and top 5 by HCD (resolution 7500) in each cycle in the LTQ (minimum intensity 1000 counts). Parent ions were then excluded from MS/MS for the next 30 s. Singly charged ions were excluded since in ESI mode peptides usually carry multiple charges. The Orbitrap was continuously recalibrated using lock-mass function [84]. Mass accuracy included an error of mass measurement within 5 ppm and did not exceed 10 ppm.

For analysis of mass spectrometry data, centroid fragment peak lists were processed with Proteome Discoverer v. 1.2 (Thermo Fisher Scientific). The search was performed with the Mascot algorithm (v. 2.4) against a database comprised of 6,692 predicted protein sequences from the source organism *C. neoformans* H99 database (*C. neoformans var. grubii* H99 Sequencing Project, Broad Institute of Harvard and MIT, http://www.broadinstitute.org/) using the following parameters: peptide mass accuracy 10 parts per million; fragment mass accuracy 0.6 Da; trypsin enzyme specificity with 1 max missed cleavages; fixed modifications - carbamidomethyl, variable modifications - methionine oxidation, deamidated N, Q and N-acetyl peptides, dimethyl (K), dimethyl (N-term), dimethyl 2H(4) (K), and dimethyl 2H(4) (N-term), ESI-TRAP fragment characteristics. Only those peptides with Ion Scores exceeding the individually calculated 99 % confidence limit (as opposed to the average limit for the whole experiment) were considered as accurately identified. The acceptance criteria for protein identification were as follows: only proteins containing at least one unique peptide with a Mascot score > 25 were considered in the dataset. Quantitative ratios were extracted from the raw data using Proteome Discoverer. Proteome Discoverer parameters – Event Detector: mass precision 4 ppm (corresponds to extracted ion chromatograms at ±12 ppm max error), S/N threshold 1; Precursor Ion Quantifier method set for ‘2 labels’ for the formaldehyde labeled samples; Quantitation Method – Ratio Calculation – Replace Missing Quantitation Values with Minimum Intensity – yes, Use Single Peak Quantitation Channels – yes, − Protein Quantification – Use All Peptides – yes.

Experimentally determined fold changes for WT and *P*_*GAL7*_*::PKA1* strains grown under Pka1-repressed (glucose-containing medium) and Pka1-induced (galactose-containing medium) conditions were converted to a log_2_ scale and the average fold change and standard deviation were used for analysis. A fold change of >10 was used as a cut-off limit for the time-point and end-point analyses. For the comparative analysis of the time-point samples, the statistical significance of the fold changes of the identified secreted proteins present under both Pka1-repressed and Pka1-induced conditions and at equivalent time points (*i.e.* 16, 48, 72, and 120 hpi) was assessed for an influence of PKA regulation using a Student’s *t-test* (*p-value* < 0.05). For the comparative analysis of the end-point samples, the statistical significance of the fold changes of the identified secreted proteins present under both Pka1-repressed and Pka1-induced conditions was evaluated using a Student’s *t-test* (*p-value* < 0.05). To confirm the statistically significant Pka1-regulated proteins identified from the end-point analysis, a multiple-hypothesis testing correction was performed on the secretome data using the Benjamini and Hochberg method with a false discovery rate of 0.05.

### Gene ontology analyses

Proteins were characterized with Gene Ontology (GO) terms using a local installation of Blast2GO [85]. Gene annotation data of the *C. neoformans* H99 reference genome were retrieved from the Broad Institute (May 2014) and a copy of the non-redundant (nr) protein database was downloaded from NCBI (May 2014) [86]. The most current associations between the nr protein database and GO terms were retrieved in May 2014 from Blast2GO. GO terms were assigned to WT proteins and filtered using default settings of the Blast2GO pipeline [85]. We performed GO term enrichment analyses for sets of proteins using hypergeometric tests and the Benjamini and Hochberg false discovery rate multiple testing correction (*p-value* < 0.05) implemented in the R packages GSEABase and GOstats. GO term categories containing singleton entries were excluded. GO categories and enrichment datasets were visualized using the R package ggplot2 [87]. For time-point analyses, GO term classification was performed on unique proteins identified under either Pka1-repressed or Pka1-induced conditions to highlight the overall influence of Pka1 regulation on the secretome profile.

### Prediction of the extracellular location of identified proteins

SignalP 4.1 (http://www.cbs.dtu.dk/services/SignalP/) was used to predict whether identified proteins were secreted based on the presence of a signal peptide. Identified protein sequences were also analyzed using Signal-3 L (http://www.csbio.sjtu.edu.cn/bioinf/Signal-3L/) and Phobius (http://phobius.sbc.su.se) to confirm results. Additionally, secreted proteins were analyzed for the presence of a glycophosphatidylinositol (GPI) anchor using GPI-SOM (http://gpi.unibe.ch).

### RNA isolation and quantitative Real-Time PCR (qRT-PCR) analysis

Cells from WT and *P*_*GAL7*_*::PKA1* strains were prepared for the examination of gene expression by overnight growth in YNB medium followed by dilution to 5.0 x 10^7^ cells/ml in 5 ml of MM + D or MM + G and incubation at 30 °C with agitation for 16 and 96 h. Samples were collected in triplicate for analysis. Cells were collected at the designated time points, flash frozen in liquid N_2_, and stored at −80 °C. Total RNA was extracted using an EZ-10 DNAaway RNA Miniprep kit (Bio Basic) according to the manufacturer’s protocol. Complementary DNA was synthesized using a Verso cDNA kit (Thermo Scientific) and used for quantitative real-time PCR (qRT-PCR). Primers were designed using Primer3 v.4.0 (http://bioinfo.ut.ee/primer3-0.4.0/) and targeted to the 3’ regions of transcripts. qRT-PCR primer sequences (see Additional file 9: Table S6). Relative gene expression was quantified using the Applied Biosystems 7500 Fast Real-time PCR system. Control genes CNAG_00483 (Actin) and CNAG_06699 (GAPDH) were used for normalization, and tested for statistical significance using the Student’s *t-test*. As a control, *PKA1* RNA expression levels under Pka1-repressed and Pka1-induced conditions in the WT and *P*_*GAL7*_*::PKA1* strains were also analyzed at various time points to confirm the regulated *PKA* expression (see Additional file 10: Figure S4).

### RNA blot analysis

To confirm qRT-PCR results, total RNA was isolated for the *P*_*GAL7*_*::PKA1* strain grown in 50 ml of MM + D or MM + G for 16 h. Briefly, cell pellets were collected and flash frozen in liquid N_2_, followed by overnight lyophilization. One milliliter of buffer 1 (2 % SDS, 68 mM Na_3_C_6_H_5_O_7_, 132 mM C_6_H_8_O_7_, 10 mM EDTA) was added to the samples, along with 600 μl of glass beads; samples were subjected to bead beating for two, 3 min intervals at power 3 (BioSpec, Mini-Beadbeater) and subsequently stored on ice. Next, 340 μl of buffer 2 (4 M NaCl, 17 mM Na_3_C_6_H_5_O_7_, 33 mM C_6_H_8_O_7_) was added and samples were inverted several times and incubated on ice for 5 min. Samples were then centrifuged at 15,000 rpm for 10 min, the supernatant fraction was collected and transferred to a new tube, one volume of isopropanol was added, and samples were mixed and incubated at room temperature for 15 min. The pellet was collected following centrifugation at 15,000 rpm for 5 min, and washing of the pellet with 70 % DEPC (Diethylpyrocarbonate)-EtOH was performed. The pellet was collected, air dried, and dissolved in 20 μl of DEPC-H_2_O. The hybridization probes were prepared with a PCR-amplified DNA fragment of CNAG_00483 (Actin) or CNAG_00396 (*PKA1*) using specific primers (see Additional file 9: Table S6) and labeled with ^32^P using an Oligolabeling kit (Amersham Biosciences). Scanned images were analyzed using a Bio-Rad ChemiDoc MP Imaging System (see Additional file 11: Figure S5).

### Multiple Reaction Monitoring (MRM) sample collection from macrophages, mouse bronchoalveolar lavage, and mouse blood

The survival rates of the WT, *pka1*Δ mutant, and *P*_*GAL7*_*::PKA1* strains during incubation with macrophages were determined and lysates were prepared for protein analysis [88]. Briefly, cells of the J774A.1 macrophage-like cell line were grown to 80 % confluence in Dulbecco’s Modified Eagle’s Medium (DMEM; Sigma) supplemented with 10 % fetal bovine serum and 2 mM L-glutamine at 37 °C and 5 % CO_2_. The macrophages were stimulated 1 h prior to infection with 150 ng/ml phorbol myristate acetate (PMA). Fungal cells were grown in YNB overnight at 30 °C, followed by inoculation in MM + D or MM + G at 5.0 x 10^7^ cells/ml. Following overnight growth, the fungal cells were washed with phosphate-buffered saline (PBS, Invitrogen) and opsonized with 0.5 μg/ml of the anti-capsule monoclonal antibody 18B7 in DMEM or DMEM supplemented with 0.20 % glucose or galactose (30 min at 37 °C). Stimulated macrophages were infected with 2.0 x 10^5^ opsonized fungal cells at a multiplicity of infection (MOI) of 1:1 for 2 h and 24 h at 37 °C and 5 % CO_2_. To measure fungal survival, macrophages containing internalized cryptococcal cells were washed thoroughly four times with PBS and then lysed in 1 ml of sterile dH_2_O for 30 min at room temperature. Lysate dilutions were plated on YPD agar and incubated at 30 °C for 48 h, at which time the resulting colony forming units (CFUs) were counted and intracellular rates of infection (%) were calculated as the ratio of the CFUs at 2 h and 24 h over the initial number of macrophages. The statistical significance of differences between WT, *pka1*Δ mutant, and *P*_*GAL7*_*::PKA1* strains were determined by unpaired *t-tests*. For proteomic analysis, lysates from infected macrophage at 24 h of incubation were collected, flash frozen in liquid N_2_ and stored at −80 °C.

Female BALB/c mice (10–12 weeks old) obtained from Charles River Laboratories (Senneville, Ontario, Canada) were used to collect bronchoalveolar lavage (BAL) and blood samples following cryptococcal infection. Three different cultures of *C. neoformans* WT cells were grown overnight in YPD at 30 °C with agitation, washed in PBS and re-suspended at 1.0 x 10^8^ cells/ml in PBS. For collection of BAL, intranasal inoculation of three mice with 100 μl of the different WT cell suspensions (1.0 x 10^7^ cells) was performed. For collection of blood samples, intravenous inoculation of three mice with 100 μl of the WT cell suspensions (1.0 x 10^7^ cells) was performed. Three mice were selected for the analysis based on established methods for studying fungal burden in mouse models of cryptococcosis [34–37]. At 48 hpi, the infected mice were euthanized by CO_2_ inhalation and 1 ml of BAL fluid and 500 μl of blood samples were collected from each mouse [89]. Mouse lavage and blood samples were flash frozen with liquid N_2_ and stored at −80 °C. Mouse assays were conducted in accordance with University of British Columbia’s Committee on Animal Care (protocol A13-0093).

### MRM sample preparation

Macrophage lysate samples were prepared as described above, followed by trypsin in-solution digestion. Samples were collected for WT and *P*_*GAL7*_*::PKA1* strains at 24 hpi in triplicate. Mouse BAL samples were prepared as described above, followed by trypsin in-solution digestion. Samples were collected at 48 hpi following WT inoculation of each of the three mice. For mouse blood samples, highly abundant proteins were removed as previously described [90]. Briefly, proteins were precipitated by the addition of two volumes of acetonitrile and 1.0 % acetic acid, followed by centrifugation at 10,000 rpm for 5 min at 4 °C. The supernatant was collected and evaporated and the residual proteins were then subjected to trypsin in-solution digestion as described above. Samples were collected at 48 hpi following WT inoculation of each of the three mice. Following trypsin digestion, all samples were desalted, concentrated, and filtered on high-capacity C18 STAGE tips.

### Peptide selection, internal standardization, and MRM method development

Skyline (v2.1) was used to build and optimize the MRM method for the relative quantification of peptides [91]. Synthesized peptides for MRM analysis were designed in-house using the following parameters: tryptic peptides, 0 max missed cleavages, minimum of seven and maximum of 25 amino acids, excluding peptides containing Met or Cys residues (if possible) and N-terminal glutamine, hydrophobicity between 10–40 (Sequence Specific Retention Calculator, http://hs2.proteome.ca/SSRCalc/SSRCalcX.html), desirable spectral intensities (GenePattern ESPPredictor, http://www.broadinstitute.org/cancer/software/genepattern/modules), and transition settings selecting for precursor charges of 2 and 3, ion charge of 1, monitoring both b and y ions. SpikeTides labeled with stable isotopes (C-term Arg U-^13^C_6_;U-^15^N_4_ or Lys U-^13^C_6_;U-^15^N_2_) were purchased from JPT Peptide Technologies GmbH (Berlin, Germany). N-terminal Arginine (R) and Lysine (K) were labeled with a stable isotope mass of 10.008269 and 8.014199, respectively. Collision energy (CE) and fragmentor voltage (FV) for each peptide was predicted utilizing Skyline software and then confirmed experimentally [92]. Doubly and triply charged precursor ions were optimized and three to five transitions were measured per peptide. The final MRM method included the monitoring of a total of 23 peptides, representing five proteins (see Additional file 12: Table S7). Stable isotope-labeled peptides were resuspended in 100 μl of 0.5 % acetic acid with agitation at room temperature. The peptides were further diluted and combined to result in final concentrations of 100 fmol/μl to 1 pmol/μl of each peptide. Five μl of the peptide mixture was injected into an Agilent 6460 Triple Quadrupole (Agilent) for data acquisition and peptide optimization.

### Mass spectrometry and data analysis for MRM

MRM assays were performed on an Agilent 6460 Triple Quadrupole coupled with Agilent 1200 Series HPLC. The instrument was operated in positive electrospray ionization mode using MassHunter Workstation Data Acquisition (v.B.04.04, Agilent). Chromatography was performed on a Large Capacity Chip with 160 nl Trap, analytical column was 150 mm x 75 μm, stationary phase for both trapping and analytical columns were Zorbax-SB C-18, 300 A and 5 μm particles (Agilent). Peptides were separated using gradient elution with a stable flow of 0.30 μl/min, beginning with 97 % buffer A (97 % dH_2_O, 3 % acetonitrile, 0.1 % formic acid (FA)) and 3 % buffer B (10 % dH_2_O, 90 % ACN, 0.1 % FA) followed by a step gradient of buffer B from 3 to 80 %, which was achieved at 10.5 min. Subsequent equilibration was performed for 4.5 min at 3 % buffer B. The column was maintained at room temperature during analysis, and the samples were kept at 4-7 °C. The MS was operating in selective reaction mode using electrospray ionization in positive ion mode, with a capillary voltage of 1850 V and a source temperature of 325 °C. Cone voltage was static, collision energies and fragmentor voltages were optimized for each compound individually (see Additional file 13: Table S8). Peak identification was performed using MassHunter Qualitative Analysis (Agilent).

Quantification of natural proteins was performed using peak areas relative to the known amounts of added isotopically-labeled synthetic peptides during a multiplexed MRM run. Natural protein levels were identified in triplicate from the following matrices: WT and *P*_*GAL7*_*::PKA1* macrophage lysate MM + D and MM + G collected at 24 hpi; BAL collected at 48 hpi from three different mice inoculated independently with the WT strain; and blood collected at 48 hpi from three different mice inoculated independently with the WT strain. Each biological sample was assayed independently in triplicate. Experimentally determined peak areas and the subsequent quantification values were converted to a log_2_ scale, and the average amount of identified peptide +/− S.D. was reported. Positive association of natural peptides to their respective isotopically-labeled peptides was determined based on co-elution patterns. For positive identification of a natural protein in a collected sample, at least one peptide with a minimum of two transitions must be identified or a minimum of two peptides with at least one transition each must be present.

### Validation of secretome data

Enzymatic activity was assayed for α-amylase and acid phosphatase. The assays were performed with kits for both enzymes according to the manufacturer’s protocol (BioVision Incorporated) (see Additional file 14: Figure S6). To confirm that proteins identified in the secretome were a result of secretion and not a product of cell lysis, a PCR was performed on secretome samples from Pka1-repressed conditions for WT strain at 96 hpi. Actin (CNAG_00483) and *PKA1* (CNAG_00396) were used as control genes for amplification (see Additional file 15: Figure S7) [48].

### Availability of supporting data

The mass spectrometry proteomics data have been deposited to the ProteomeXchange Consortium [93] via the PRIDE partner repository [94] with the dataset identifier PXD002731 and the PASSEL partner repository with the dataset identifier PASS00736.

### Ethics statement

Mouse assays were conducted in accordance with University of British Columbia’s Committee on Animal Care (protocol A13-0093).

## Additional files

Additional file 1: Table S1. Quantitative proteomic analysis of the secretome of *C. neoformans* at 16, 48, 72, and 120 hpi under Pka1-repressed (glucose-containing medium) conditions. (DOCX 94 kb) Additional file 2: Table S2. Quantitative proteomic analysis of the secretome of *C. neoformans* at 16, 48, 72, and 120 hpi under Pka1-induced (galactose-containing medium) conditions. (DOCX 99 kb) Additional file 3: Table S3. Proteins identified in the secretome of *C. neoformans* from both the 96 hpi (end-point) samples and the time course (16, 48, 72, 120 hpi) samples prepared in Pka1-repressed (glucose-containing medium) and Pka1-induced (galactose-containing medium) conditions. (DOCX 94 kb) Additional file 4: Table S4. Quantitative proteomic analysis of the secretome of *C. neoformans* at 96 hpi under Pka1-repressed (glucose-containing medium) conditions. (DOCX 92 kb) Additional file 5: Table S5. Quantitative proteomic analysis of the secretome of *C. neoformans* at 96 hpi under Pka1-induced (galactose-containing medium) conditions. (DOCX 82 kb) Additional file 6: Figure S1. Enrichment of genes represented in the secretome for cells grown under Pka1-repressed (D) and Pka1-induced (G) conditions compared to all genes present in the WT strain. Enrichment based on GO terms associated with cellular compartment (CC). (TIFF 275 kb) Additional file 7: Figure S2. Comparison of GO terms classifications of cellular components from the identified secreted proteins grown under Pka1-repressed and Pka1-induced conditions compared to proteins represented in the Fungal Secretome Knowledgebase. (TIFF 1613 kb) Additional file 8: Figure S3. Comparison of GO terms classifications of molecular function from the identified secreted proteins grown under Pka1-repressed and Pka1-induced conditions compared to proteins represented in the Fungal Secretome Knowledgebase. (TIFF 1149 kb) Additional file 9: Table S6. Primers sequences used for amplification of cDNA for qRT-PCR analysis and genomic DNA for cell lysis control PCR. (DOCX 21 kb) Additional file 10: Figure S4. RNA expression levels of *PKA1* measured by qRT-PCR in *P*_*GAL7*_ *::PKA1* and WT strains, grown in Pka1-repressed (glucose-containing medium) and Pka1-induced (galactose-containing medium) conditions at 4, 16, 48 and 96 hpi. Samples evaluated in triplicate, values reported as average log_2_ fold change ± S.D. Actin and GAPDH were used as controls and *PKA1* expression was assessed under the different growth conditions. The results show an up-regulation of *PKA1* mRNA under Pka1-inducible conditions. (TIFF 610 kb) Additional file 11: Figure S5. RNA expression levels of *PKA1* measured by Northern blot as a method for validating the qRT-PCR results. RNA was extracted from *P*_*GAL7*_ *::PKA1* cells grown under Pka1-repressed (glucose-containing medium) and Pka1-induced (galactose-containing medium) conditions 16 hpi. Actin was used as a control and *PKA1* expression was assessed under the different growth conditions. The results show an up-regulation of *PKA1* mRNA under Pka1-inducible conditions. (TIFF 342 kb) Additional file 12: Table S7. Proteins selected for Multiple Reaction Monitoring assays and their respective isotopically-labeled synthetic peptides (DOCX 87 kb) Additional file 13: Table S8. Isotopically-labeled peptides monitored during MRM multiplex assay. (DOCX 112 kb) Additional file 14: Figure S6. Enzyme activity for A) Amylase and B) Acid phosphatase in the WT and *P*_*GAL7*_ *::PKA1* strains of *C. neoformans* under Pka1-repressed (glucose-containing medium) and Pka1-induced (galactose-containing medium) conditions. Values are reported as an average ± standard deviation for three independent replicates. For statistical analysis, a Student’s *t-test* was performed and a line denotes *p-value* < 0.05. (TIFF 489 kb) Additional file 15: Figure S7. PCR to confirm absence of cellular factors due to cell lysis in secretome of *C. neoformans*. PCR for the A) Actin and B) PKA1 genes were performed using either *C. neoformans* WT genomic DNA (6 pg to 100 ng) or WT supernatant samples (250 ng or 1000 ng of total protein). Control lane contains 6 pg of gDNA spiked into 250 ng of supernatant. (TIFF 1093 kb)

## Abbreviations

AIDS: Acquired immune deficiency syndrome
BAL: Bronchoalveolar lavage
cAMP: cyclic-adenosine monophosphate
CID: Collision induced dissociation
CFU: Colony forming units
DEPC: Diethylpyrocarbonate
DMEM: Dulbecco’s modified eagle medium
FunSecKB: Fungal Secretome KnowledgeBase
GO: Gene ontology
GPCR: G-protein coupled receptor
GPI: Glycophosphatidylinositol
HCD: Higher-energy collision dissociation
HIV: Human immunodeficiency virus
hpi: Hours post inoculation
HPLC: High performance liquid chromatography
MM (D/G): Minimal media (glucose/galactose)
MRM: Multiple reaction monitoring
NEM: N-ethylmaleimide
PBS: Phosphate buffered saline
PKA: Protein kinase A
PMA: Phorbol myristate acetate
qRT-PCR: Quantitative real time – polymerase chain reaction
SID: Stable isotope dilutions
STAGE: Stop and go extraction
WT: Wild-type
YPD: Yeast peptone dextrose
