# Supplementary material for: Secretome profiling of Cryptococcus neoformans reveals regulation of a subset of virulence-associated proteins and potential biomarkers by protein kinase A
Source: BMC Microbiol. 2015 Oct 9;15:206. doi: 10.1186/s12866-015-0532-3 (PMC4600298; doi:10.1186/s12866-015-0532-3)
Supplement: Additional file 1: Table S1. — Quantitative proteomic analysis of the secretome of C. neoformans at 16, 48, 72, and 120 hpi under Pka1-repressed (glucose-containing medium) conditions. (DOCX 94 kb) [file 12866_2015_532_MOESM1_ESM.docx]

**Table S1**: Quantitative proteomic analysis of the secretome of *C. neoformans* at 16, 48, 72, and 120 hpi under Pka1-repressed (glucose-containing medium) conditions.

| **Accession number** | **Protein Name** | **#Pep^a^** | **Peptide sequence** | **Charge state** | **Time point** | **Fold change ± S.D.^b^** |
| --- | --- | --- | --- | --- | --- | --- |
| CNAG_02189 | Alpha-amylase | 2 | sVYQVIVDR | 2 | 16; 120 | 0.298±0.314; 0.695±0.733 |
|  |  |  | vLIEDSQk | 2 |  |  |
| CNAG_06125 | Translation elongation factor 1 alpha | 4 | qTVAVGVIk | 2 | 16 | 0.135±0.145 |
|  |  |  | iGGIGTVPVGR | 2 |  |  |
|  |  |  | fAPTNVTTEVk | 2 |  |  |
|  |  |  | sGDAAIVk | 2 |  |  |
| CNAG_00799 | Cellulase | 6 | dSGAWSGNk | 2 | 16; 120 | 0.302±0.320; 0.151±0.001 |
|  |  |  | fSLSTLENR | 2 |  |  |
|  |  |  | iVDSDGNEVILR | 2 |  |  |
|  |  |  | tVQNADGSWR | 2 |  |  |
|  |  |  | mDWAIAEAk | 2 |  |  |
|  |  |  | dAVGEIWkk | 2 |  |  |
| CNAG_04245 | Chitinase | 3 | lVSSGHAAGk | 3 | 16; 48; 120 | 0.448±0.007; 0.826±1.019; 0.075;0.064 |
|  |  |  | aQFAAQAGLR | 2 |  |  |
|  |  |  | sSESGQIVTYDDTESmNLk | 2 |  |  |
| CNAG_06501 | 1,3-beta-glucanosyltransferase | 2 | sVGSSALVGYAAVDGEPDFR | 2 | 16; 120 | 0.573±0.383; 1.475±0.172 |
|  |  |  | yLYDESGNR | 2 |  |  |
| CNAG_01239 | Chitin deacetylase | 1 | vEDDLYSPPGEk | 2 | 16; 72 | 0.906±0.310; 3.415±0.568 |
| CNAG_03525 | Trehalase | 2 | aFETLGR | 2 | 16 | 0.404±0.095 |
|  |  |  | tFVDkPTAk | 2 |  |  |
| CNAG_01137 | Aconitase | 3 | fINYQR | 2 | 48 | 0.529±0.733 |
|  |  |  | aGSALNmmASAAk | 2 |  |  |
|  |  |  | fSDPSGYELPAk | 2 |  |  |
| CNAG_06101 | Eukaryotic ADP/ATP carrier | 7 | gNTANVIR | 2 | 16; 120 | 0.075±0.060; 0.791± 1.091 |
|  |  |  | gVAGAGVLSLYDk | 2 |  |  |
|  |  |  | gAGANILR | 2 |  |  |
|  |  |  | tAAAPIER | 2 |  |  |
|  |  |  | sMFDAGSQIIAk | 2 |  |  |
|  |  |  | lLVQNQDEmIk | 2 |  |  |
|  |  |  | lATPYk | 2 |  |  |
| CNAG_02974 | Voltage-dependent ion-selective channel | 10 | aSAGVSVDTTR | 2 | 16; 48; 120 | 0.223±0.089; 0.184±0.223; 1.436; 0.135 |
|  |  |  | gPTFTADTVVGR | 2 |  |  |
|  |  |  | sTAGNVSLEVGAk | 2 |  |  |
|  |  |  | lNEPTAGQAAHk | 3 |  |  |
|  |  |  | sSSDLLLk | 2 |  |  |
|  |  |  | TDAISGDIEGk | 2 |  |  |
|  |  |  | tQLELENQIAk | 2 |  |  |
|  |  |  | dYPIQGTSLEVk | 2 |  |  |
|  |  |  | tLTPSNVAFk | 2 |  |  |
|  |  |  | fDLATTLNPAk | 2 |  |  |
| CNAG_03465 | Laccase | 2 | eYTFDITk | 2 | 16; 120 | 0.637±0.651; 1.043±1.256 |
|  |  |  | aLASPDGYER | 2 |  |  |
|  |  | 3 | ySAIINTSEGk | 2 |  |  |
| CNAG_02944 | Acid phosphatase | 2 | yPTSGAGPSTFAAk | 2 | 16 | 0.789±0.404 |
|  |  |  | lADATAQDGGFTAk | 2 |  |  |
| CNAG_00581 | Endopeptidase | 1 | nAVGFAESk | 2 | 16 | 0.083±0.063 |
| CNAG_03072 | Phosphopyruvate hydratase | 4 | eIDDLLIk | 2 | 16 | 0.073±0.052 |
|  |  |  | vIAPALIDSk | 2 |  |  |
|  |  |  | gTTAPELHSSk | 3 |  |  |
|  |  |  | aEVPSGASTGAHEAVELR | 3 |  |  |
| CNAG_00483 | Actin | 3 | gILTLk | 2 | 48 | 0.134±0.027 |
|  |  |  | aGFAGDDAPR | 2 |  |  |
|  |  |  | eITALAPSSMk | 2 |  |  |
| CNAG_02030 | Glyoxal oxidase | 5 | TGLSASANER | 2 | 16; 48 | 0.278±0.324; 0.242±0.063 |
|  |  |  | tILYDYNTk | 2 |  |  |
|  |  |  | tTTDLPDmPYATR | 2 |  |  |
|  |  |  | iSPDNDNPQYEDDDYmFEGR | 2 |  |  |
|  |  |  | sQGmGGWLQmTGk | 2 |  |  |
| CNAG_00407 | Glyoxal oxidase | 4 | eGLGMTTQER | 2 | 120 | 1.320±1.619 |
|  |  |  | wQEGGDELTmTSk | 2 |  |  |
|  |  |  | dNPNVPNDFmDTDGGAAIR | 2 |  |  |
|  |  |  | tGFSTHAmNmGQR | 3 |  |  |
| CNAG_06267 | Rds1 protein | 2 | fSDAEFEQYGINAEQR | 2 | 16; 120 | 0.336±0.303; 0.860±0.444 |
|  |  |  | qQmIFR | 2 |  |  |
| CNAG_02864 | Predicted protein | 2 | qScASTcLQR | 2 | 16; 48 | 0.346±0.056; 1.965±0.909 |
|  |  |  | dTPASSTTLAYVGcVSDDSVSSLTSGSASTNAEAR | 3 |  |  |
| CNAG_00776 | Immunoreactive mannoprotein MP88 | 1 | vIPDGVLTAVHFVk | 3 | 16; 120 | 0.627±0.214; 3.337±3.493 |
| CNAG_04753 | Lactonohydrolase | 4 | iSLLEAAAVQk | 2 | 16; 48 | 1.031±0.631; 1.250±0.996 |
|  |  |  | aTDEVFFVQNAGAk | 2 |  |  |
|  |  |  | nAQVINPk | 2 |  |  |
|  |  |  | qFNSLNDISVNPR | 2 |  |  |
| CNAG_05312 | Conserved hypothetical protein | 1 | vIPPGAITGAHFVk | 3 | 16 | 0.119±0.030 |
| CNAG_01047 | Conserved hypothetical protein | 3 | ISHTTAEVR | 2 | 16; 72 | 0.279±0.070; 0.928±1.034 |
|  |  |  | dAGSNEIVFYR | 2 |  |  |
|  |  |  | sTcYEIk | 2 |  |  |
| CNAG_03492 | Conserved hypothetical protein | 2 | tVEVIVQSYPLAR | 2 | 16; 72 | 0.506±0.400; 0.640±0.199 |
|  |  |  | eVVcLk | 2 |  |  |
| CNAG_05893 | Conserved hypothetical protein | 3 | gGcDDSTIR | 2 | 16; 48; 120 | 0.894±0.103; 1.609±1.380; 1.641±0.953 |
|  |  |  | eIMVAYIk | 2 |  |  |
|  |  |  | eLYDIVYVVNPLk | 2 |  |  |
|  |  |  | wETQmPYALGLk | 2 |  |  |
| CNAG_00587 | Conserved hypothetical protein | 2 | qTWNYSAQNk | 2 | 16; 120 | 1.275±0.018; 3.449±1.211 |
|  |  |  | eGSGQINQNPYVIVk | 2 |  |  |
| CNAG_05595 | Conserved hypothetical protein | 1 | tPSDLGNSR | 2 | 120 | 1.257±0.379 |

^a^Number of peptides identified for the protein

^b^Fold change is reported as the average quantification for *P_GAL7_::PKA1* vs. WT.
