# Supplementary material for: Secretome profiling of Cryptococcus neoformans reveals regulation of a subset of virulence-associated proteins and potential biomarkers by protein kinase A
Source: BMC Microbiol. 2015 Oct 9;15:206. doi: 10.1186/s12866-015-0532-3 (PMC4600298; doi:10.1186/s12866-015-0532-3)
Supplement: Additional file 2: Table S2. — Quantitative proteomic analysis of the secretome of C. neoformans at 16, 48, 72, and 120 hpi under Pka1-induced (galactose-containing medium) conditions. (DOCX 99 kb) [file 12866_2015_532_MOESM2_ESM.docx]

**Table S2**: Quantitative proteomic analysis of the secretome of *C. neoformans* at 16, 48, 72, and 120 hpi under Pka1-induced (galactose-containing medium) conditions.

| **Accession number** | **Protein Name** | **#Pep^a^** | **Sequence** | **Charge state** | **Time point** | **Fold change ± S.D.^b^** |
| --- | --- | --- | --- | --- | --- | --- |
| CNAG_02189 | Alpha-amylase | 3 | tLSSSLHAR | 3 | 16; 48; 72 | 1.096±1.270; 0.807±0.494; 1.289±0.420 |
|  |  |  | sVYQVIVDR | 2 |  |  |
|  |  |  | mTVSTPSESTLLVAk | 2 |  |  |
| CNAG_06840 | Translation elongation factor 2 | 8 | gGGQIIPTAR | 2 | 16 | 0.212±0.271 |
|  |  |  | tNVLAVNIR | 2 |  |  |
|  |  |  | sGTLTTSETAHNmR | 3 |  |  |
|  |  |  | aGIIASSk | 2 |  |  |
|  |  |  | sAEAGVER | 2 |  |  |
|  |  |  | sDPVVGYR | 2 |  |  |
|  |  |  | kSDPVVGYR | 2 |  |  |
|  |  |  | aEPLGEELTR | 2 |  |  |
| CNAG_06125 | Translation elongation factor 1 alpha | 6 | qTVAVGVIk | 2 | 16 | 0.379±0.517 |
|  |  |  | qLIVAcNk | 2 |  |  |
|  |  |  | STTTGHLIYk | 2 |  |  |
|  |  |  | iGGIGTVPVGR | 2 |  |  |
|  |  |  | fAPTNVTTEVk | 2 |  |  |
|  |  |  | yQVTVIDAPGHR | 3 |  |  |
| CNAG_04245 | Chitinase | 3 | lVSSGHAAGk | 3 | 16; 48 | 0.286±0.296; 0.603±0.017 |
|  |  |  | aQFAAQAGLR | 2 |  |  |
|  |  |  | sSESGQIVTYDDTESMNLk | 2 |  |  |
| CNAG_06313 | Phosphoglucomutase | 1 | tIVVGGDGR | 2 | 16 | .365±.465 |
| CNAG_06501 | 1,3-beta-glucanosyltransferase | 2 | yLYDESGNR | 2 | 16; 48 | 0.896±0.169; 0.872±0.066 |
|  |  |  | sVGSSALVGYAAVDGEPDFR | 2 |  |  |
| CNAG_06291 | Deacetylase | 2 | vEDAFVk | 2 | 16 | 1.018±0.761 |
|  |  |  | yFRPPYGNINDNVLk | 3 |  |  |
| CNAG_01239 | Chitin deacetylase | 1 | vEDDLYSPPGEk | 2 | 16 | 1.350±0.515 |
| CNAG_00799 | Cellulase | 3 | dSGAWSGNk | 2 | 48 | 0.422±0.248 |
|  |  |  | mDWAIAEAk | 2 |  |  |
|  |  |  | iVDSDGNEVILR | 2 |  |  |
| CNAG_03266 | Malate dehydrogenase | 2 | gADIVVIPAGVPR | 2 | 16 | 0.232±0.252 |
|  |  |  | iQFGGDEVVk | 2 |  |  |
| CNAG_01984 | Transaldolase | 7 | lLEPAIQYAk | 2 | 48 | 1.784±1.617 |
|  |  |  | fSFDTQATINk | 2 |  |  |
|  |  |  | gGSVDVQSENAmDR | 2 |  |  |
|  |  |  | aNPDTTYTAENDPGVQSVQk | 3 |  |  |
|  |  |  | sQDDLPk | 2 |  |  |
|  |  |  | lLDELNk | 2 |  |  |
|  |  |  | aTGTTVVADTGDFTSIDk | 2 |  |  |
| CNAG_02974 | Voltage-dependent ion-selective channel | 10 | sAILTAIYk | 2 | 16; 72 | 0.466±0.645; 18.390±19.742 |
|  |  |  | aSAGVSVDTTR | 2 |  |  |
|  |  |  | gPTFTADTVVGR | 2 |  |  |
|  |  |  | sTAGNVSLEVGAk | 2 |  |  |
|  |  |  | lNEPTAGQAAHk | 3 |  |  |
|  |  |  | tQLELENQIAk | 2 |  |  |
|  |  |  | dYPIQGTSLEVk | 2 |  |  |
|  |  |  | aTVDLFk | 2 |  |  |
|  |  |  | sSSDLLLk | 2 |  |  |
|  |  |  | fDLATTLNPAk | 2 |  |  |
| CNAG_05750 | ATPase alpha subunit | 6 | sQHGALLEk | 3 | 16; 72 | 0.482±0.463; 7.436±8.164 |
|  |  |  | sVDSLVPIGR | 2 |  |  |
|  |  |  | eIEEEMkk | 2 |  |  |
|  |  |  | vVDALGNPIDGk | 2 |  |  |
|  |  |  | sALAVDTILNQk | 2 |  |  |
|  |  |  | iAGASAGGDVQETGR | 2 |  |  |
| CNAG_06699 | Glyceraldehyde-3-phosphate dehydrogenase | 3 | aSESPELk | 2 | 16 | .294±0.396 |
|  |  |  | kASESPELk | 2 |  |  |
|  |  |  | gAAANIIPSSTGAAk | 2 |  |  |
| CNAG_03072 | Phosphopyruvate hydratase | 4 | eIDDLLIk | 2 | 72 | 7.938±2.612 |
|  |  |  | vIAPALIDSk | 2 |  |  |
|  |  |  | gTTAPELHSSk | 3 |  |  |
|  |  |  | aEVPSGASTGAHEAVELR | 3 |  |  |
| CNAG_03465 | Laccase | 5 | eSFSAPR | 2 | 16; 48 | 0.870±1.038; 0.815±0.251 |
|  |  |  | eYTFDITk | 2 |  |  |
|  |  |  | aLASPDGYER | 2 |  |  |
|  |  |  | ySAIINTSEGk | 2 |  |  |
|  |  |  | eFSQSHVFNSQR | 3 |  |  |
| CNAG_02944 | Acid phosphatase | 3 | gDLDFLNk | 2 | 48 | 0.485±0.134 |
|  |  |  | yPTSGAGPSTFAAk | 2 |  |  |
|  |  |  | lADATAQDGGFTAk | 2 |  |  |
| CNAG_00919 | Carboxypeptidase D | 2 | vLPQVIEATNR | 2 | 16; 48 | 3.814±3.024; 3.217±1.643 |
|  |  |  | gDLSADPIQk | 2 |  |  |
| CNAG_01750 | Chaperone | 2 | dAGAIAGLDVLR | 2 | 16 | 0.251±0.339 |
|  |  |  | aTAGDTHLGGEDFDNR | 3 |  |  |
| CNAG_06150 | Heat-shock protein 90 | 7 | eTLQQNk | 2 | 16 | 0.545±0.592 |
|  |  |  | lGIHEDATNR | 3 |  |  |
|  |  |  | aDLVNNLGTIAk | 2 |  |  |
|  |  |  | eLISNSSDALDk | 2 |  |  |
|  |  |  | eGLELEETPEEk | 2 |  |  |
|  |  |  | ikETTTENEELNk | 3 |  |  |
|  |  |  | nPQDVTQEEYASFYk | 2 |  |  |
| CNAG_00334 | Heat shock protein | 4 | tkLDISDDAR | 3 | 16 | 1.335±0.629 |
|  |  |  | aQITIQNSVGR | 2 |  |  |
|  |  |  | vDDIVLVGGSTR | 2 |  |  |
|  |  |  | aVVTVPAYFNDSQR | 3 |  |  |
| CNAG_06746 | Histone h2b | 2 | lILPGELSk | 2 | 16 | 0.246±0.328 |
|  |  |  | qVHPDTGISNk | 3 |  |  |
| CNAG_04021 | 60S ribosomal protein L26 | 1 | aSSLGLASDR | 2 | 16 | 1.444±1.275 |
| CNAG_04114 | 40S ribosomal protein S0 | 2 | fVDIAIPTNNk | 2 | 16 | 1.925±1.065 |
|  |  |  | yGSFTGAQAIAGR | 2 |  |  |
| CNAG_06605 | Ribosomal protein S2 | 1 | gTGIVAAPASk | 2 | 72 | 20.635±22.855 |
| CNAG_02864 | Predicted protein | 2 | qScASTcLQR | 2 | 16 | 0.259±0.183 |
|  |  |  | dTPASSTTLAYVGcVSDDSVSSLTSGSASTNAEAR | 3 |  |  |
| CNAG_02943 | Cytoplasmic protein | 3 | sHFDTVSR | 2 | 16 | 0.464±0.357 |
|  |  |  | eGPITQAVR | 2 |  |  |
|  |  |  | aIGAVNNTPmQVSSk | 2 |  |  |
| CNAG_02030 | Glyoxal oxidase | 6 | TGLSASANER | 2 | 16; 48; 72; 120 | 0.600±0.672; 0.350±0.144; 1.586±1.696; 0.970±0.973 |
|  |  |  | tILYDYNTk | 2 |  |  |
|  |  |  | gGFNTHAmGFGQk | 3 |  |  |
|  |  |  | sQGmGGWLQmTGk | 2 |  |  |
|  |  |  | tTTDLPDMPYATR | 2 |  |  |
|  |  |  | iSPDNDNPQYEDDDYmFEGR | 2 |  |  |
| CNAG_04753 | Lactonohydrolase | 2 | nAQVINPk | 2 | 16; 48 | 0.751±0.413; 1.055±0.546 |
|  |  |  | qFNSLNDISVNPR | 2 |  |  |
| CNAG_06267 | Rds1 protein | 2 | qQmIFR | 2 | 16; 48; 72; 120 | 0.756±0.222; 0.748±0.234; 1.057±0.036; 5.106±4.588 |
|  |  |  | FSDAEFEQYGINAEQR | 2 |  |  |
| CNAG_00776 | Immunoreactive mannoprotein MP88 | 1 | vIPDGVLTAVHFVk | 3 | 16; 48; 72 | 0.994±1.193; 1.598±0.521; 1.494±0.177 |
| CNAG_02850 | Glucan endo-1,3-alpha-glucosidase agn1 | 2 | vSLSEGSISGSISR | 2 | 48 | 0.500±0.176 |
|  |  |  | yANAAAQATYEGk | 2 |  |  |
| CNAG_04291 | Glycosyl-hydrolase | 1 | vLVYSATAPDGYR | 2 | 120 | 1.347±0.232 |
| CNAG_01047 | Conserved hypothetical protein | 3 | sTcYEIk | 2 | 16; 48; 72; 120 | 0.471±0.272; 0.285±0.077; 0.719±0.432; 1.468±0.279 |
|  |  |  | dAGSNEIVFYR | 2 |  |  |
|  |  |  | iLDYAVEk | 2 |  |  |
| CNAG_03492 | Conserved hypothetical protein | 1 | tVEVIVQSYPLAR | 2 | 16; 48; 72 | 0.799±0.030; 0.456±0.032; 1.180±0.431 |
|  |  |  | eVVcLk | 2 |  |  |
| CNAG_00587 | Conserved hypothetical protein | 2 | qTWNYSAQNk | 2 | 16; 48; 72 | 1.620±0.371; 2.327±1.740; 3.126±0.550 |
|  |  |  | eGSGQINQNPYVIVk | 2 |  |  |
| CNAG_00586 | Conserved hypothetical protein | 1 | yEGGLYFYSGDYQGQNAETQAR | 2 | 16 | 2.195±0.678 |
| CNAG_05893 | Conserved hypothetical protein | 3 | eIMVAYIk | 2 | 48; 72; 120 | 1.676±1.639; 1.087±0.049; 1.413±0.348 |
|  |  |  | gGcDDSTIRk | 3 |  |  |
|  |  |  | wETQmPYALGLk | 2 |  |  |
| CNAG_05312 | Conserved hypothetical protein | 1 | vIPPGAITGAHFVk | 3 | 48; 120 | 8.287±9.682; 8.860±3.671 |
| CNAG_05595 | Conserved hypothetical protein | 1 | tPSDLGNSR | 2 | 72 | 2.350±0.922 |

^a^Number of peptides identified for the protein

^b^Fold change is reported as the average quantification for *P_GAL7_::PKA1* vs. WT.
