# Supplementary material for: Secretome profiling of Cryptococcus neoformans reveals regulation of a subset of virulence-associated proteins and potential biomarkers by protein kinase A
Source: BMC Microbiol. 2015 Oct 9;15:206. doi: 10.1186/s12866-015-0532-3 (PMC4600298; doi:10.1186/s12866-015-0532-3)
Supplement: Additional file 3: Table S3. — Proteins identified in the secretome of C. neoformans from both the 96 hpi (end-point) samples and the time course (16, 48, 72, 120 hpi) samples prepared in Pka1-repressed (glucose-containing medium) and Pka1-induced (galactose-containing medium) conditions. (DOCX 94 kb) [file 12866_2015_532_MOESM3_ESM.docx]

**Table S3:** Proteins identified in the secretome of *C. neoformans* from both the 96 hpi (end-point) samples and the time course (16, 48, 72, 120 hpi) samples prepared in Pka1-repressed (glucose-containing medium) and Pka1-induced (galactose-containing medium) conditions.

|  |  | **Fold change^a^**  **(Pka1-repressed)** | | **Fold change^a^**  **(Pka1-induced)** | |
| --- | --- | --- | --- | --- | --- |
| **Accession number** | **Protein name** | **End-point** | **Time-point** | **End-point** | **Time-point** |
| CNAG_02189 | α-Amylase | NS | < 0.5, NS | NS | NS |
| CNAG_06125 | Translation elongation factor 1 α | > 2.0 | < 0.5 | < 0.5 | < 0.5 |
| CNAG_01239 | Chitin deacetylase | > 2.0 | NS, > 2.0 | > 2.0 | NS |
| CNAG_04245 | Chitinase | . | . | NS | < 0.5, NS |
| CNAG_06501 | 1,3-β-glucanosyltransferase | NS | NS | NS | NS |
| CNAG_05750 | ATPase α subunit | . | . | NS | < 0.5, > 2.0 |
| CNAG_06101 | Eukaryotic ADP/ATP carrier | > 2.0 | < 0.5, NS | . | . |
| CNAG_02944 | Acid phosphatase | NS | NS | < 0.5 | < 0.5 |
| CNAG_03072 | Phosphopyruvate hydratase | > 2.0 | < 0.5 | . | . |
| CNAG_03465 | Laccase | NS | NS | NS | NS |
| CNAG_00919 | Carboxypeptidase D | . | . | > 2.0 | > 2.0 |
| CNAG_01750 | Chaperone | . | . | > 2.0 | < 0.5 |
| CNAG_00407 | Glyoxal oxidase | NS | NS | . | . |
| CNAG_02030 | Glyoxal oxidase | NS | < 0.5 | . | . |
| CNAG_04753 | Lactonohydrolase | . | . | > 2.0 | NS |
| CNAG_06267 | Rds1 protein | > 2.0 | < 0.5, NS | > 2.0 | NS, > 2.0 |
| CNAG_01047 | Conserved hypothetical protein | > 2.0 | < 0.5, NS | . | . |
| CNAG_05312 | Conserved hypothetical protein | < 0.5 | < 0.5 | > 2.0 | > 2.0 |
| CNAG_05893 | Conserved hypothetical protein | NS | NS | NS | NS |

^a^Fold change presented as NS (not significant, value is > 0.5 and < 2.0), < 0.5, and > 2.0. Discrepancies may be attributed to the different precipitation methods used and to the collection of proteins at different time points. If protein was identified at more than one time point, the data is presented sequentially.
