# Supplementary material for: Secretome profiling of Cryptococcus neoformans reveals regulation of a subset of virulence-associated proteins and potential biomarkers by protein kinase A
Source: BMC Microbiol. 2015 Oct 9;15:206. doi: 10.1186/s12866-015-0532-3 (PMC4600298; doi:10.1186/s12866-015-0532-3)
Supplement: Additional file 4: Table S4. — Quantitative proteomic analysis of the secretome of C. neoformans at 96 hpi under Pka1-repressed (glucose-containing medium) conditions. (DOCX 92 kb) [file 12866_2015_532_MOESM4_ESM.docx]

**Table S4**: Quantitative proteomic analysis of the secretome of *C. neoformans* at 96 hpi under Pka1-repressed (glucose-containing medium) conditions.

| **Accession number** | **Protein Name** | **#Pep^a^** | **Sequence** | **Charge state** |
| --- | --- | --- | --- | --- |
| CNAG_02189 | Alpha-amylase | 2 | sVYQVIVDR | 2 |
|  |  |  | fESFVTDASLIk | 2 |
| CNAG_06125 | Translation elongation factor 1 alpha | 2 | qTVAVGVIk | 2 |
|  |  |  | fAPTNVTTEVk | 2 |
| CNAG_02860 | Endo-1,3(4)-beta-glucanase | 2 | sGIAAWFFQR | 2 |
|  |  |  | gSAFTEAFWEVASVk | 3 |
| CNAG_06501 | 1,3-beta-glucanosyltransferase | 2 | dLPYLQQLGVNAVR | 2 |
|  |  |  | sVGSSALVGYAAVDGEPDFR | 3 |
| CNAG_05799 | Chitin deacetylase | 3 | sIINcLGk | 2 |
|  |  |  | iLTEYAPk | 2 |
|  |  |  | tTFFVVGSR | 2 |
| CNAG_06291 | Deacetylase | 3 | yFRPPYGNINDNVLk | 3 |
|  |  |  | gTFFLNGANYVcIYDk | 2 |
|  |  |  | aLYDAGHTLGSHTWSHADLTQLDESGINDELSk | 4 |
| CNAG_01239 | Chitin deacetylase | 2 | vEDDLYSPPGEk | 2 |
|  |  |  | nVADAFNMEWYLNSGk | 2 |
| CNAG_03225 | Malate dehydrogenase | 4 | vFGITTLDVVR | 2 |
|  |  |  | dDLFNTNASIVR | 2 |
|  |  |  | eALTGAEIVIIPAGVPR | 2 |
|  |  |  | iNPVGQLSAEEQELLk | 3 |
| CNAG_07561 | Phosphogluconate dehydrogenase | 2 | dFFGAHTFR | 3 |
|  |  |  | gDIIIDGGNSHYPDSIR | 3 |
| CNAG_01920 | Polyubiquitin | 2 | eSTLHLVLR | 3 |
|  |  |  | tITLEVESSDTIDNVk | 3 |
| CNAG_05918 | F0F1 ATP synthase subunit beta | 2 | lVLEVAQHLGENTVR | 3 |
|  |  |  | iPSAVGYQPTLSTDmGGmQER | 3 |
| CNAG_05750 | ATPase alpha subunit | 5 | sVDSLVPIGR | 2 |
|  |  |  | iAGASAGGDVQETGR | 2 |
|  |  |  | tGQIVDVPVGPGLLGR | 2 |
|  |  |  | vLTIGDGIAR | 2 |
|  |  |  | sFTELLk | 2 |
| CNAG_06101 | Eukaryotic ADP/ATP carrier | 2 | gAGANILR | 2 |
|  |  |  | dEGLASLWR | 2 |
| CNAG_01890 | 5-methyltetrahydropteroyltriglutamate-homocysteine S-methyltransferase | 3 | aIQVDEPAIR | 2 |
|  |  |  | sYGYSNEIGPGVYDIHSPR | 3 |
|  |  |  | lHLELGVVSGR | 3 |
| CNAG_02944 | Acid phosphatase | 5 | gDLDFLNk | 2 |
|  |  |  | gFLEEFVAR | 2 |
|  |  |  | fMINDAVLPLDk | 2 |
|  |  |  | lGAELLTPFGR | 2 |
|  |  |  | lQNFELGVTFR | 2 |
| CNAG_03072 | Phosphopyruvate hydratase | 3 | vIAPALIDSk | 2 |
|  |  |  | gNPTVEVDLHTEk | 3 |
|  |  |  | aEVPSGASTGAHEAVELR | 3 |
| CNAG_01019 | Cu/Zn superoxide dismutase | 3 | sLVVHASTDDLGk | 3 |
|  |  |  | iISLYGPHSIIGR | 3 |
|  |  |  | hVGDLGNIQTNScGAAQLDFSDk | 3 |
| CNAG_03465 | Laccase | 5 | eGDAFWLR | 2 |
|  |  |  | fISATAHPMYR | 3 |
|  |  |  | gSPAPPQGDAILInGR | 2 |
|  |  |  | eYTFDITk | 2 |
|  |  |  | aLASPDGYER | 2 |
| CNAG_00919 | Carboxypeptidase D | 4 | vLPQVIEATNR | 2 |
|  |  |  | gDLSADPIQk | 2 |
|  |  |  | nFQDLFGIk | 2 |
|  |  |  | tSEFLIk | 2 |
| CNAG_01727 | Hsc70-4 | 4 | fDLSGIPPAPR | 2 |
|  |  |  | dAGAIAGLDVLR | 2 |
|  |  |  | nGLESYAYSLk | 2 |
|  |  |  | aVVTVPAYFNDSQR | 3 |
| CNAG_06095 | Ribosomal protein L13 | 4 | eSNAFTTLR | 2 |
|  |  |  | eALSLGISVDPR | 2 |
|  |  |  | aGDATGDDLTAHITR | 3 |
|  |  |  | dSIPLPASYTAEAPR | 2 |
| CNAG_01653 | Cytokine inducing-glycoprotein | 2 | aQITDFETSPVAFAFPEPR | 2 |
|  |  |  | tSYPMSGGEIALVQqTDAQNVNILWTSESDPTR | 3 |
|  |  |  | fHSFSTYSNSIR | 3 |
| CNAG_00407 | Glyoxal oxidase | 3 | dNPNVPNDFmDTDGGAAIR | 2 |
|  |  |  | tILYDLDAQQETPLPDmPYAVR | 3 |
|  |  |  | smGQFIYmPDGk | 2 |
| CNAG_04291 | Glycosyl-hydrolase | 3 | vLVYSATAPDGYR | 2 |
|  |  |  | wTFQEEVYYFSSNPR | 2 |
|  |  |  | hDSIPTAIEVLGQNADk | 2 |
| CNAG_02030 | Glyoxal oxidase | 6 | gGFNTHAmGFGQk | 3 |
|  |  |  | sQGMGGWLQMTGk | 2 |
|  |  |  | tTTDLPDMPYATR | 2 |
|  |  |  | aENNPITINGAYGTHPAWATEYDIETNEYR | 3 |
|  |  |  | tGLSASANER | 2 |
|  |  |  | iSPDNDNPQYEDDDYmFEGR | 3 |
| CNAG_06267 | Rds1 protein | 2 | fSDAEFEQYGINAEQR | 2 |
|  |  |  | sLIEFmADQEVGHATLISNmLGASGAPk | 3 |
| CNAG_05312 | Conserved hypothetical protein | 2 | vIPPGAITGAHFVk | 3 |
|  |  |  | iGNVEQIVVSYcLk | 2 |
| CNAG_03007 | Conserved hypothetical protein | 4 | aYENHVAR | 3 |
|  |  |  | aQAEQALDQSGHY | 2 |
|  |  |  | LSHELIGGAAGFEAMk | 2 |
|  |  |  | eIIAGLAAAEVDk | 2 |
| CNAG_01562 | Conserved hypothetical protein | 2 | hITLTnTNNGk | 2 |
|  |  |  | akPTTYDEGYLESYDSYHAR | 3 |
| CNAG_05893 | Conserved hypothetical protein | 4 | eImVAYIk | 2 |
|  |  |  | wETQMPYALGLk | 3 |
|  |  |  | eLYDIVYVVNPLk | 2 |
|  |  |  | nTNLPFLFLQPSmASSALcTPcTR | 3 |
| CNAG_01047 | Conserved hypothetical protein | 2 | dAGSNEIVFYR | 2 |
|  |  |  | iLDYAVEk | 2 |
| CNAG_00588 | Conserved hypothetical protein | 2 | tWDINPGSGSVILHGTNFALDAGTGSDNNEGVk | 3 |
|  |  |  | iWTSYPGLFQQTWFLTGDQR | 3 |
| CNAG_03223 | Conserved hypothetical protein | 2 | dTVWQSNSPQTVEWTAVDTDPR | 3 |
|  |  |  | vESPNkDTVWQSNSPQTVEWTAVDTDPR | 4 |
| CNAG_00586 | Conserved hypothetical protein | 1 | yEGGLYFYSGDYQGQNAETQAR | 2 |

^a^Number of peptides identified for the protein
