# Supplementary material for: Secretome profiling of Cryptococcus neoformans reveals regulation of a subset of virulence-associated proteins and potential biomarkers by protein kinase A
Source: BMC Microbiol. 2015 Oct 9;15:206. doi: 10.1186/s12866-015-0532-3 (PMC4600298; doi:10.1186/s12866-015-0532-3)
Supplement: Additional file 5: Table S5. — Quantitative proteomic analysis of the secretome of C. neoformans at 96 hpi under Pka1-induced (galactose-containing medium) conditions. (DOCX 82 kb) [file 12866_2015_532_MOESM5_ESM.docx]

**Table S5**: Quantitative proteomic analysis of the secretome of *C. neoformans* at 96 hpi under Pka1-induced (galactose-containing medium) conditions.

| **Accession number** | **Protein Name** | **#Pep^a^** | **Sequence** | **Charge state** |
| --- | --- | --- | --- | --- |
| CNAG_02189 | Alpha-amylase | 2 | sVYqVIVDR | 2 |
|  |  |  | fESFVTDASLIk | 2 |
| CNAG_06125 | Translation elongation factor 1 alpha | 2 | qTVAVGVIk | 2 |
|  |  |  | fAPTNVTTEVk | 2 |
| CNAG_04245 | Chitinase | 2 | lVSSGHAAGk | 3 |
|  |  |  | aQFAAQAGLR | 2 |
| CNAG_06501 | 1,3-beta-glucanosyltransferase | 2 | dLPYLQQLGVNAVR | 2 |
|  |  |  | sVGSSALVGYAAVDGEPDFR | 2 |
| CNAG_01239 | Chitin deacetylase | 2 | vEDDLYSPPGEk | 2 |
|  |  |  | nVADAFNMEWYLNSGk | 2 |
| CNAG_02860 | Endo-1,3(4)-beta-glucanase | 2 | sGIAAWFFQR | 2 |
|  |  |  | gSAFTEAFWEVASVk | 3 |
| CNAG_01920 | Polyubiquitin | 4 | lIFAGk | 2 |
|  |  |  | mQIFVk | 2 |
|  |  |  | eSTLHLVLR | 2 |
|  |  |  | tLSDYNIQk | 2 |
| CNAG_05750 | ATPase alpha subunit | 4 | sVDSLVPIGR | 2 |
|  |  |  | iAGASAGGDVQETGR | 2 |
|  |  |  | tGQIVDVPVGPGLLGR | 2 |
|  |  |  | vLTIGDGIAR | 2 |
| CNAG_02944 | Acid phosphatase | 4 | gFLEEFVAR | 2 |
|  |  |  | fMINDAVLPLDk | 2 |
|  |  |  | gDLDFLNk | 2 |
|  |  |  | lGAELLTPFGR | 2 |
| CNAG_01019 | Cu/Zn superoxide dismutase | 3 | aVVVLk | 2 |
|  |  |  | iISLYGPHSIIGR | 3 |
|  |  |  | sLVVHASTDDLGk | 3 |
| CNAG_03465 | Laccase | 8 | ySAIINTSEGk | 2 |
|  |  |  | fISATAHPMYR | 3 |
|  |  |  | eFSQSHVFNSQR | 3 |
|  |  |  | gSPAPPQGDAILINGR | 2 |
|  |  |  | eGDAFWLR | 2 |
|  |  |  | aLASPDGYER | 2 |
|  |  |  | iTIDNHPLEVVETDGTAVYGPTVHEISIAPGER | 4 |
|  |  |  | eYTFDITk | 2 |
| CNAG_00919 | Carboxypeptidase D | 3 | vLPQVIEATNR | 2 |
|  |  |  | aTTEEEIAQDFIk | 2 |
|  |  |  | gDLSADPIQk | 2 |
|  |  |  | tSEFLIk | 2 |
| CNAG_01750 | Chaperone | 4 | vEIIANDQGNR | 2 |
|  |  |  | aTAGDTHLGGEDFDNR | 3 |
|  |  |  | dAGAIAGLDVLR | 2 |
|  |  |  | nGLESYAYSLk | 2 |
| CNAG_04291 | Glycosyl-hydrolase (347 aa) | 3 | vLVYSATAPDGYR | 2 |
|  |  |  | hDSIPTAIEVLGQNADk | 2 |
|  |  |  | wTFQEEVYYFSSNPR | 3 |
| CNAG_02030 | Glyoxal oxidase | 6 | tGLSASANER | 2 |
|  |  |  | sQGMGGWLQMTGk | 2 |
|  |  |  | tTTDLPDMPYATR | 2 |
|  |  |  | gVPSMAEFIMVGNGR | 2 |
|  |  |  | iSPDNDNPQYEDDDYmFEGR | 3 |
|  |  |  | gGFNTHAMGFGQk | 3 |
| CNAG_06267 | Rds1 protein | 2 | fSDAEFEQYGINAEQR | 2 |
|  |  |  | sLIEFmADQEVGHATLISNMLGASGAPk | 3 |
| CNAG_01653 | Cytokine inducing-glycoprotein | 3 | fHSFSTYSNSIR | 3 |
|  |  |  | aQITDFETSPVAFAFPEPR | 2 |
|  |  |  | tSYPmSGGEIALVQQTDAQNVNILWTSESDPTR | 3 |
| CNAG_04753 | Lactonohydrolase | 2 | nAQVINPk | 2 |
|  |  |  | qFNSLNDISVNPR | 2 |
| CNAG_06109 | Conserved hypothetical protein | 3 | gTPIADQFmGGHSSGAR | 3 |
|  |  |  | gAAAAGAGVGAIGGAGVAAGLAGR | 3 |
|  |  |  | dLDTGGPHSLVYQESTGk | 3 |
| CNAG_05893 | Conserved hypothetical protein | 3 | eImVAYIk | 2 |
|  |  |  | wETQMPYALGLk | 2 |
|  |  |  | eLYDIVYVVNPLk | 2 |
| CNAG_05312 | Conserved hypothetical protein | 2 | vIPPGAITGAHFVk | 3 |
|  |  |  | iGNVEQIVVSYcLk | 2 |

^a^Number of peptides identified for the protein
