# Supplementary figures and images for: Secretome profiling of Cryptococcus neoformans reveals regulation of a subset of virulence-associated proteins and potential biomarkers by protein kinase A
Source: BMC Microbiol. 2015 Oct 9;15:206. doi: 10.1186/s12866-015-0532-3 (PMC4600298; doi:10.1186/s12866-015-0532-3)

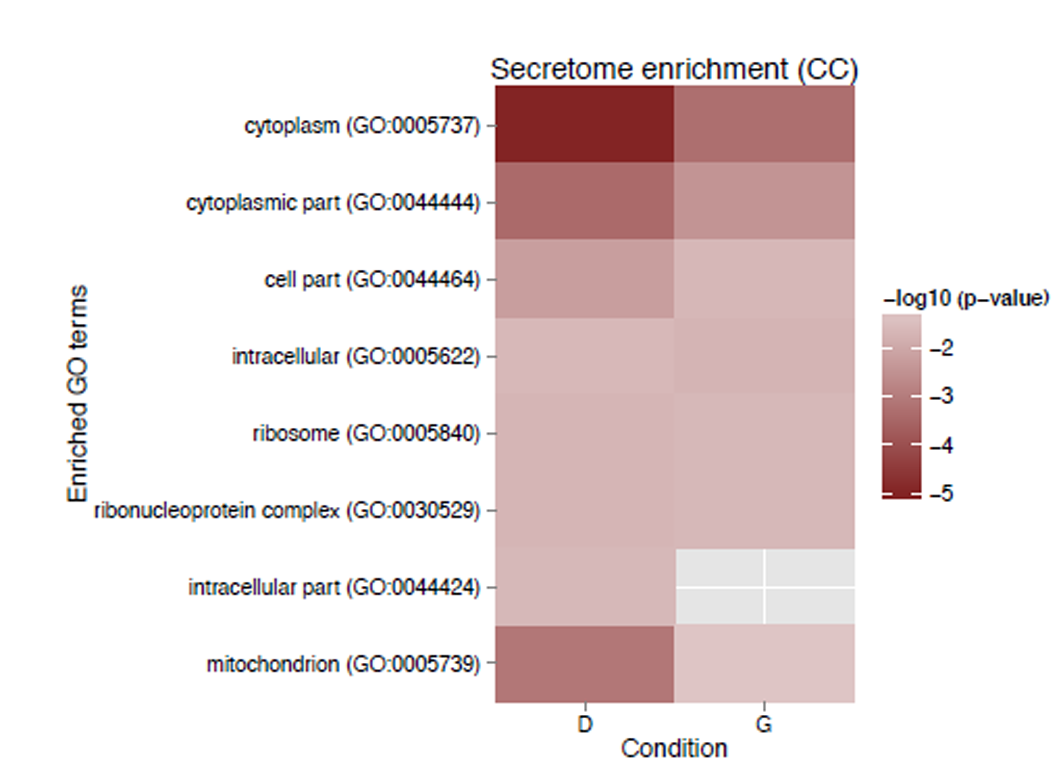

Supplement: Additional file 6: Figure S1. — Enrichment of genes represented in the secretome for cells grown under Pka1-repressed (D) and Pka1-induced (G) conditions compared to all genes present in the WT strain. Enrichment based on GO terms associated with cellular compartment (CC). (TIFF 275 kb) [file 12866_2015_532_MOESM6_ESM.tif]

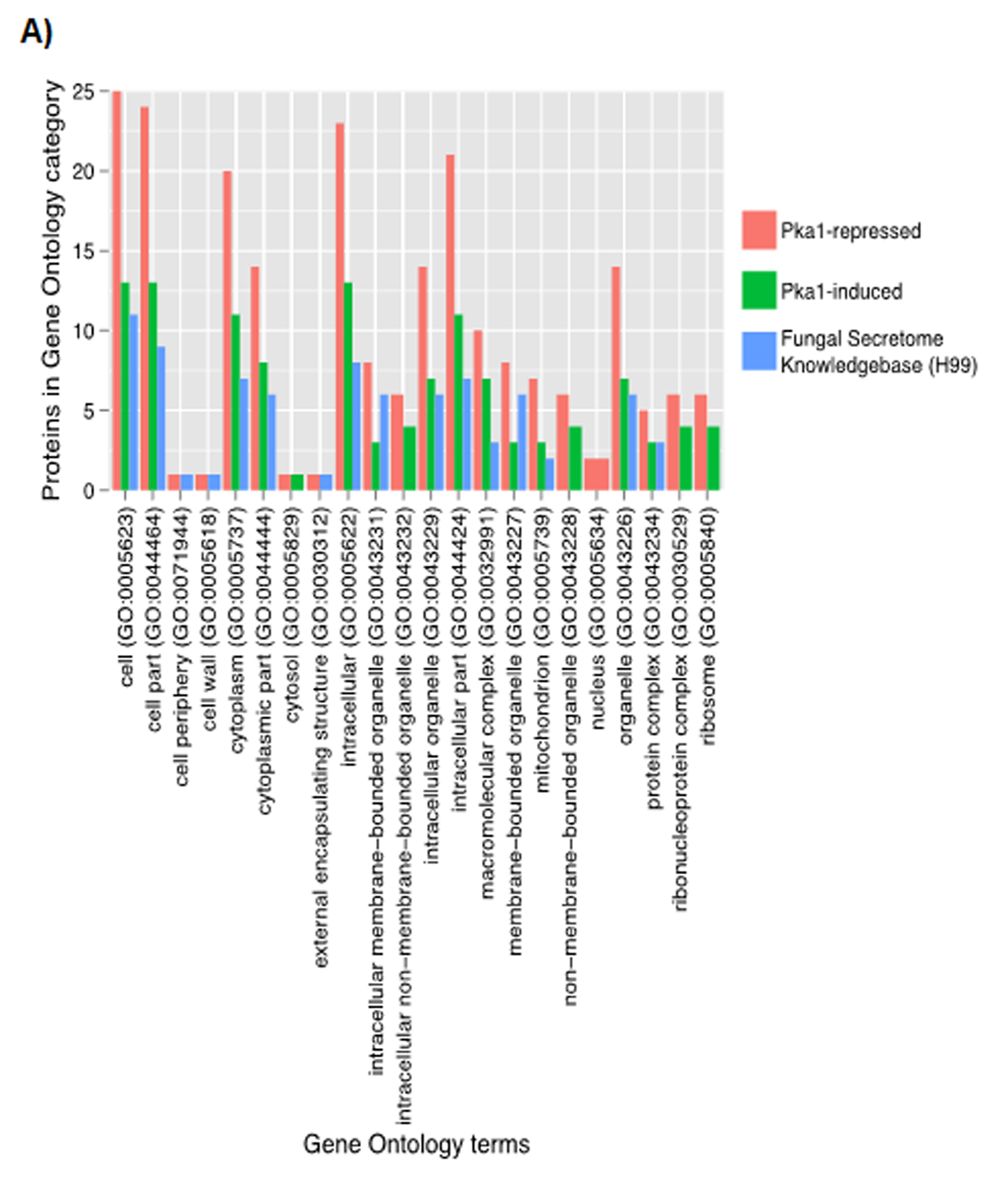

Supplement: Additional file 7: Figure S2. — Comparison of GO terms classifications of cellular components from the identified secreted proteins grown under Pka1-repressed and Pka1-induced conditions compared to proteins represented in the Fungal Secretome Knowledgebase. (TIFF 1613 kb) [file 12866_2015_532_MOESM7_ESM.tif]

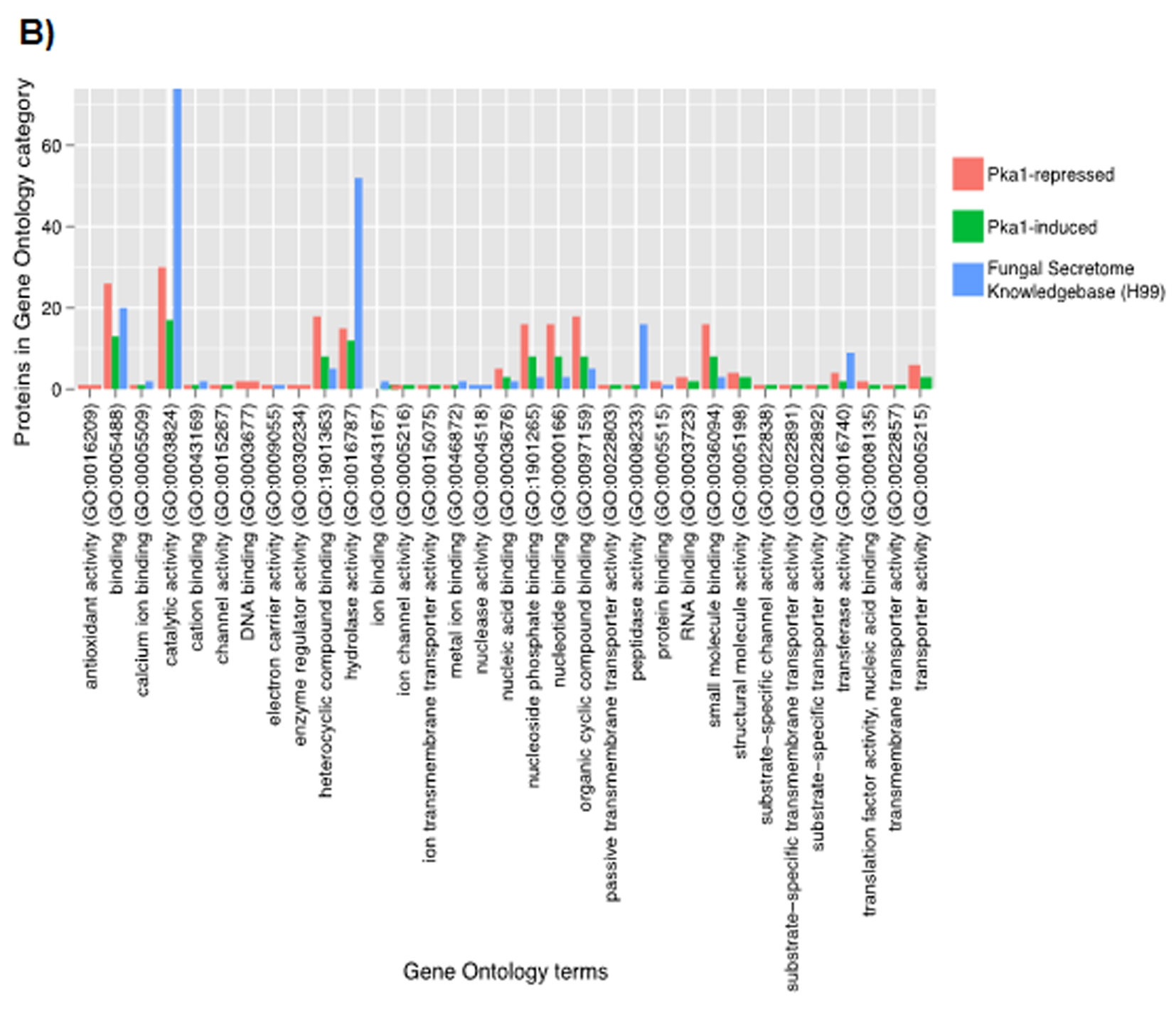

Supplement: Additional file 8: Figure S3. — Comparison of GO terms classifications of molecular function from the identified secreted proteins grown under Pka1-repressed and Pka1-induced conditions compared to proteins represented in the Fungal Secretome Knowledgebase. (TIFF 1149 kb) [file 12866_2015_532_MOESM8_ESM.tif]

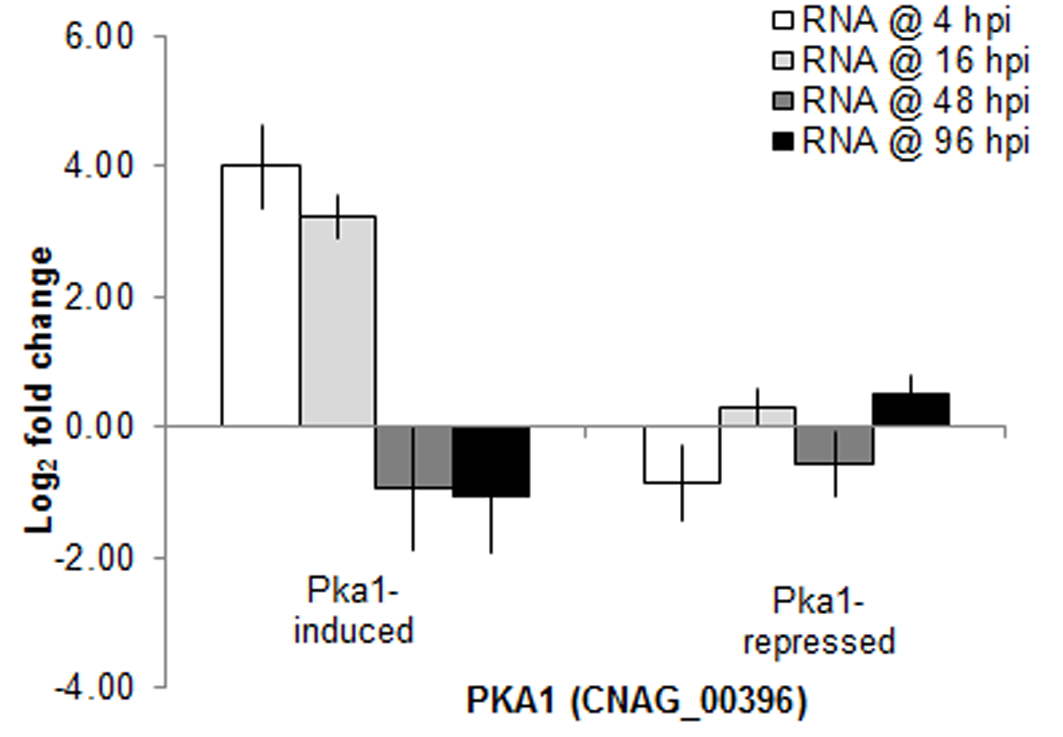

Supplement: Additional file 10: Figure S4. — RNA expression levels of PKA1 measured by qRT-PCR in P GAL7 ::PKA1 and WT strains, grown in Pka1-repressed (glucose-containing medium) and Pka1-induced (galactose-containing medium) conditions at 4, 16, 48 and 96 hpi. Samples evaluated in triplicate, values reported as average log2 fold change ± S.D. Actin and GAPDH were used as controls and PKA1 expression was assessed under the different growth conditions. The results show an up-regulation of PKA1 mRNA under Pka1-inducible conditions. (TIFF 610 kb) [file 12866_2015_532_MOESM10_ESM.tif]

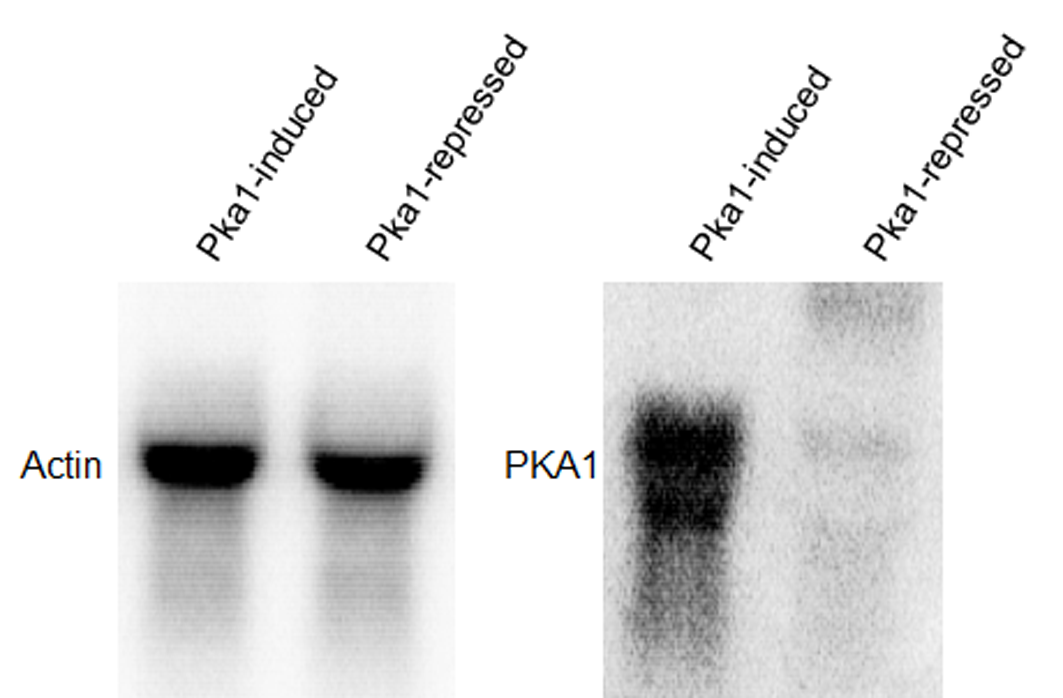

Supplement: Additional file 11: Figure S5. — RNA expression levels of PKA1 measured by Northern blot as a method for validating the qRT-PCR results. RNA was extracted from P GAL7 ::PKA1 cells grown under Pka1-repressed (glucose-containing medium) and Pka1-induced (galactose-containing medium) conditions 16 hpi. Actin was used as a control and PKA1 expression was assessed under the different growth conditions. The results show an up-regulation of PKA1 mRNA under Pka1-inducible conditions. (TIFF 342 kb) [file 12866_2015_532_MOESM11_ESM.tif]

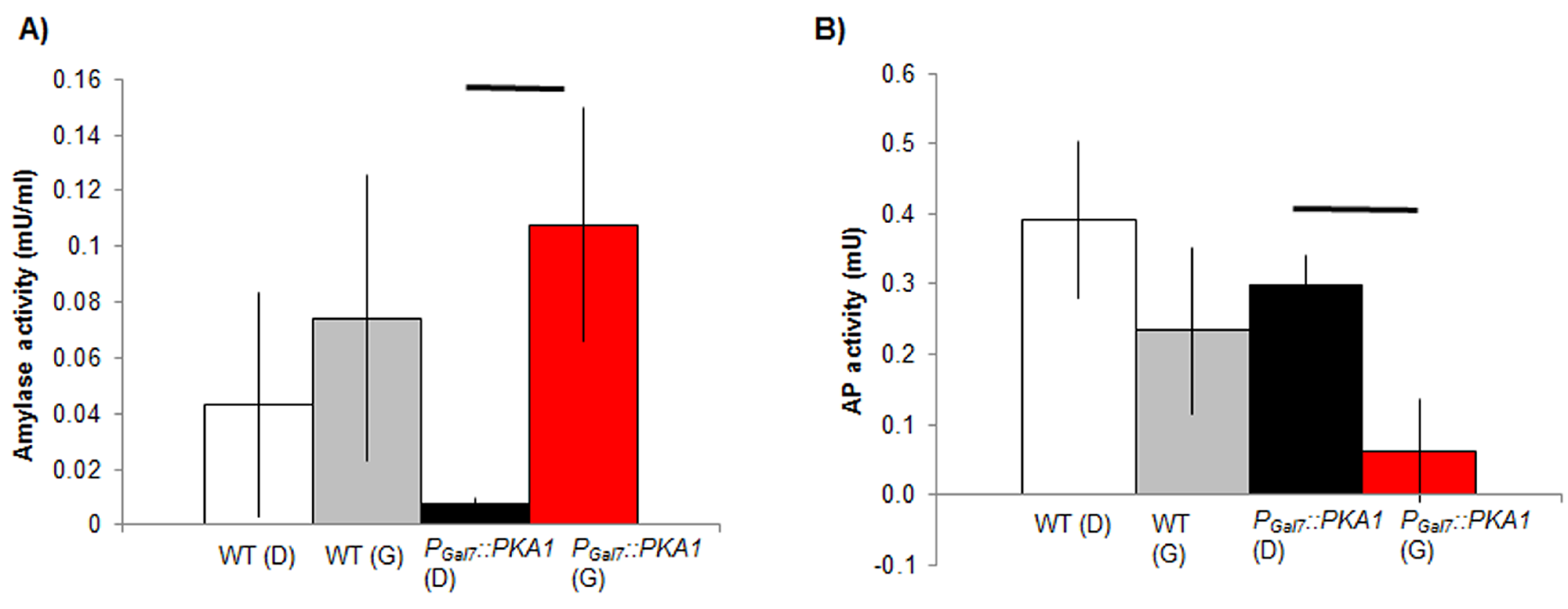

Supplement: Additional file 14: Figure S6. — Enzyme activity for A) Amylase and B) Acid phosphatase in the WT and P GAL7 ::PKA1 strains of C. neoformans under Pka1-repressed (glucose-containing medium) and Pka1-induced (galactose-containing medium) conditions. Values are reported as an average ± standard deviation for three independent replicates. For statistical analysis, a Student’s t-test was performed and a line denotes p-value < 0.05. (TIFF 489 kb) [file 12866_2015_532_MOESM14_ESM.tif]

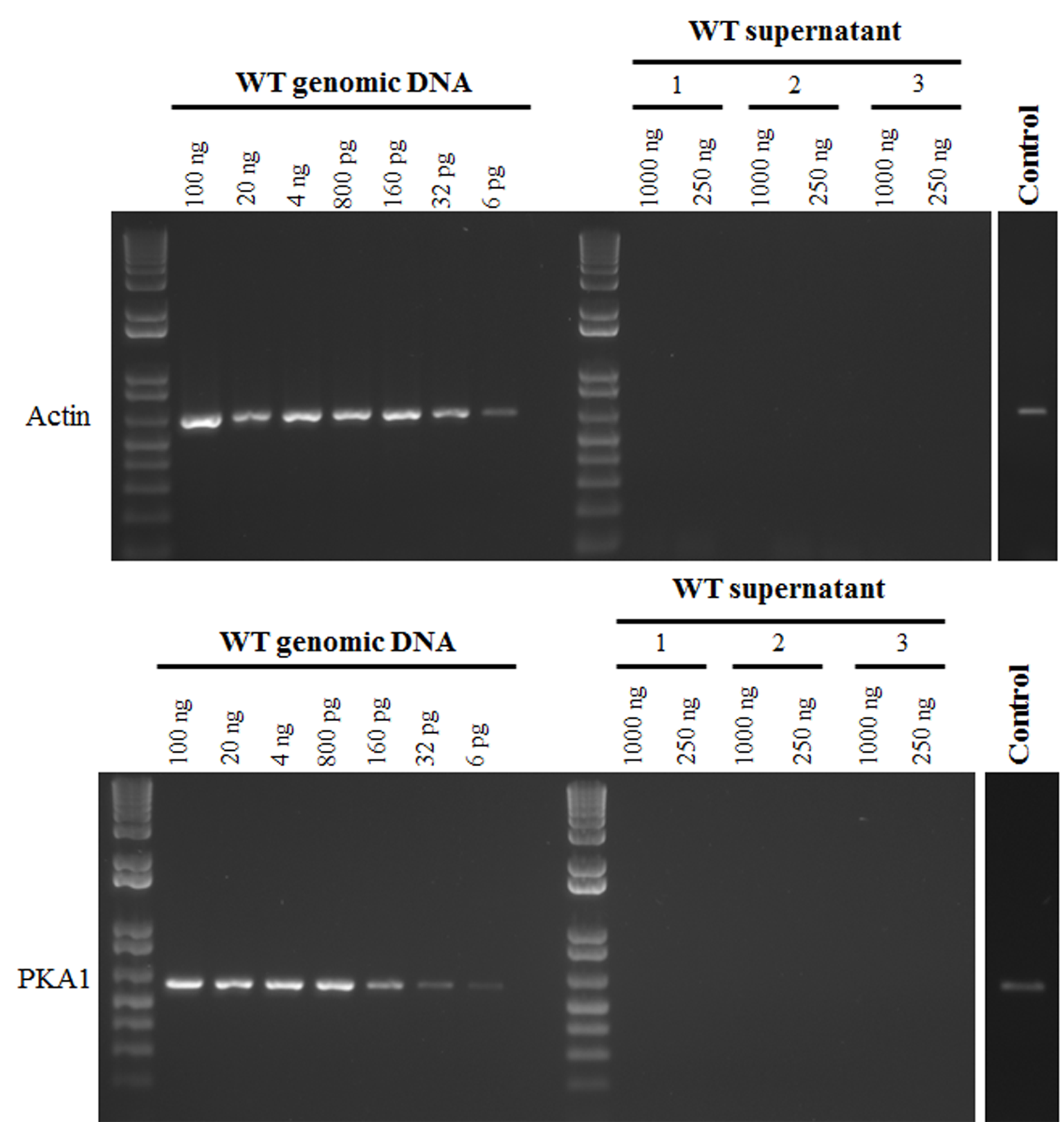

Supplement: Additional file 15: Figure S7. — PCR to confirm absence of cellular factors due to cell lysis in secretome of C. neoformans. PCR for the A) Actin and B) PKA1 genes were performed using either C. neoformans WT genomic DNA (6 pg to 100 ng) or WT supernatant samples (250 ng or 1000 ng of total protein). Control lane contains 6 pg of gDNA spiked into 250 ng of supernatant. (TIFF 1093 kb) [file 12866_2015_532_MOESM15_ESM.tif]
