# Supplementary material for: Secretome profiling of Cryptococcus neoformans reveals regulation of a subset of virulence-associated proteins and potential biomarkers by protein kinase A
Source: BMC Microbiol. 2015 Oct 9;15:206. doi: 10.1186/s12866-015-0532-3 (PMC4600298; doi:10.1186/s12866-015-0532-3)
Supplement: Additional file 9: Table S6. — Primers sequences used for amplification of cDNA for qRT-PCR analysis and genomic DNA for cell lysis control PCR. (DOCX 21 kb) [file 12866_2015_532_MOESM9_ESM.docx]

**Table S6:** Primer sequences

| **Gene Description** | **Primer Name** | **Primer Sequence** |
| --- | --- | --- |
| Protein Kinase A | CNAG_00396_qrt-F | GCTGGCAAAGTCCGCTATCC |
| (Pka1) | CNAG_00396_qrt-R | CCGGAATTTCGCGCCTGTAC |
| α-Amylase | CNAG_02189_qrt-F | GTTCTTCCGCTTCCAACACC |
|  | CNAG_02189_qrt-R | CCTTTTTGGCTGTCCTCGAT |
| Acid Phosphatase | CNAG_02944rtf | CAGCAACTCGACCTACTTCC |
|  | CNAG_02944rtr | ACCTGCGAAGCCACAAAC |
| Glyoxal oxidase | CNAG_00407rtf | ATGGTTGGCTCTGGGAAGATTG |
|  | CNAG_00407rtr | TTTGCGATGCAGATGTCCCTTG |
| Cytokine-inducing glycoprotein | CNAG_01653rtf | GGAGCCGACACCTACTACTAC |
| (Cig1) | CNAG_01653rtr | AGCCTCCAGAGGTGCTTTC |
| Hypothetical | CNAG_05312rtf | ACATCGTACACGGCCTACGC |
|  | CNAG_05312rtr | GTAGTTGCCGCAGCATTGGAG |
| Actin | CNAG_00483_qrt-F | AAGGAGATCACCGCCCTTG |
|  | CNAG_00483_qrt-R | GGGACCAGACTCGTCGTATTC |
| GAPDH | CNAG_06699_qrt-F | GGACCTCGTTTGTCGCATTG |
|  | CNAG_06699_qrt-R | AGCCTGGGCATCGAAGATAG |
| Protein Kinase A | CNAG_00396_F-Nblot | GCACCCTTCCATTAACTCCA |
| (Pka1) | CNAG_00396_R-Nblot | CCCGTACCAACATCTCTCGT |
| Actin | CNAG_00483_F-Nblot | GGTATGTGCAAGGCTGGTTT |
|  | CNAG_00483_R-Nblot | AACCACGCTCCATGAGAATC |
