# Supplementary material for: Secretome profiling of Cryptococcus neoformans reveals regulation of a subset of virulence-associated proteins and potential biomarkers by protein kinase A
Source: BMC Microbiol. 2015 Oct 9;15:206. doi: 10.1186/s12866-015-0532-3 (PMC4600298; doi:10.1186/s12866-015-0532-3)
Supplement: Additional file 12: Table S7. — Proteins selected for Multiple Reaction Monitoring assays and their respective isotopically-labeled synthetic peptides (DOCX 87 kb) [file 12866_2015_532_MOESM12_ESM.docx]

**Table S7:** Proteins selected for Multiple Reaction Monitoring assays and their respective isotopically-labeled synthetic peptides.

| **Gene Identification** | **Peptide Identification** | **Peptide Sequence^a^** | **Quantity^b^ (fmol)** |
| --- | --- | --- | --- |
| Cytokine-inducing glycoprotein | Cig1-1 | FHSFSTYSNSIR | 1090 |
| (CNAG_01653) | Cig1-2 | AQITDFETSPVAFAFPEPR | 1090 |
|  | Cig1-3 | GFSASTADDAPCGGFDPVNR | 218 |
|  | Cig1-4 | FTFTAAMAASAASAISVQR | 1090 |
| Glyoxal Oxidase | GO-1 | IIMVGSGK | 109 |
| (CNAG_00407) | GO-2 | YLELNSTYTK | 218 |
|  | GO-3 | AFSQQQAR | 1090 |
|  | GO-4 | DVTFEK | 218 |
|  | GO-5 | EGLGMTTQER | 218 |
| α-Amylase | AA-1 | FFTALNAVR | 109 |
| (CNAG_02189) | AA-2 | SVYQVIVDR | 1090 |
|  | AA-3 | FESFVTDASLIK | 109 |
|  | AA-4 | SAAGNASSTFYTDK | 109 |
|  | AA-5 | DLVSNYTIDAIR | 109 |
|  | AA-6 | VLIEDSQK | 218 |
| Acid phosphatase | AP-1 | GDLDFLNK | 109 |
| (CNAG_02944) | AP-2 | GFLEEFVAR | 1090 |
|  | AP-3 | LGAELLTPFGR | 1090 |
|  | AP-4 | LQNFELGVTFR | 218 |
| Hypothetical protein | Hyp-1 | IGNVEQIVVSYCLK | 1090 |
| (CNAG_05312) | Hyp-2 | VSYVQVTGVGDLTK | 218 |
|  | Hyp-3 | VIPPGAITGAHFVK | 218 |
|  | Hyp-4 | NGYGTR | 1090 |

^a^N-terminal Arginine (R) and Lysine (K) residues were labeled with a stable isotope.

^b^Quantity refers to the amount of each isotopically-labeled peptide spiked into the samples.
