# Supplementary material for: Secretome profiling of Cryptococcus neoformans reveals regulation of a subset of virulence-associated proteins and potential biomarkers by protein kinase A
Source: BMC Microbiol. 2015 Oct 9;15:206. doi: 10.1186/s12866-015-0532-3 (PMC4600298; doi:10.1186/s12866-015-0532-3)
Supplement: Additional file 13: Table S8. — Isotopically-labeled peptides monitored during MRM multiplex assay. (DOCX 112 kb) [file 12866_2015_532_MOESM13_ESM.docx]

**Table S8:** Isotopically-labeled peptides monitored during MRM multiplex assay.

|  |  | **Isotopically-labeled peptide** | | **Natural peptide** | |  | | |
| --- | --- | --- | --- | --- | --- | --- | --- | --- |
|  |  | **Transitions^a^** | | **Transitions^a^** | |  |  |  |
| **Gene Identification** | **Peptide Sequence** | **Q1** | **Q3** | **Q1** | **Q3** | **Ion type** | **CE^b^** | **FV^c^** |
| Cig1 | FHSFSTYSNSIR | 728.3480 | 937.4110 | 723.3440 | 927.4030 | y8 | 24.3 | 260 |
| (CNAG_01653) |  |  | 1171.5117 |  | 1161.5035 | y10 | 23.3 | 260 |
|  |  |  | 1084.4797 |  | 1074.4715 | y9 | 24.3 | 260 |
|  |  | 485.9012 | 850.3793 | 482.5651 | 840.3710 | y7 | 7.1 | 220 |
|  |  |  | 749.3316 |  | 739.3233 | y6 | 10.1 | 220 |
|  |  |  | 586.2683 |  | 576.2600 | y5 | 9.1 | 220 |
|  | AQITDFETSPVAFAFPEPR | 1067.0300 | 1328.6373 | 1062.0258 | 1318.6290 | y12 | 39.6 | 280 |
|  |  |  | 1227.5896 |  | 1217.5813 | y11 | 37.6 | 280 |
|  |  |  | 1140.5576 |  | 1130.5493 | y10 | 37.6 | 280 |
|  |  | 711.6891 | 944.4364 | 708.3530 | 934.4281 | y8 | 16.4 | 220 |
|  |  |  | 873.3993 |  | 863.3910 | y7 | 19.4 | 220 |
|  |  |  | 726.3309 |  | 716.3226 | y6 | 15.4 | 220 |
|  | GFSASTADDAPCGGFDPVNR | 1025.9435 | 495.2000 | 1020.9394 | 485.1917 | y4 | 31.5 | 280 |
|  | modification: carbamidation |  | 1128.4000 |  | 1118.3917 | y10 | 31.5 | 280 |
| α-Amylase | FFTALNAVR | 524.7940 | 901.4629 | 519.7902 | 891.4547 | y8 | 13.9 | 240 |
| (CNAG_02189) |  |  | 754.3945 |  | 744.3863 | y7 | 12.9 | 240 |
|  |  |  | 653.3469 |  | 643.3386 | y6 | 10.9 | 240 |
|  |  |  | 582.3097 |  | 572.3015 | y5 | 12.9 | 240 |
|  | SVYQVIVDR | 544.8020 | 902.4470 | 539.7982 | 892.4387 | y7 | 14.0 | 240 |
|  |  |  | 739.3836 |  | 729.3754 | y6 | 14.0 | 240 |
|  |  |  | 611.3251 |  | 601.3168 | y5 | 13.0 | 240 |
|  |  |  | 512.2566 |  | 502.2484 | y4 | 14.0 | 240 |
|  | FESFVTDASLIK | 682.8630 | 1217.6003 | 678.8559 | 1209.5861 | y11 | 19.1 | 260 |
|  |  |  | 1088.5578 |  | 1080.5436 | y10 | 18.1 | 260 |
|  |  |  | 1001.5257 |  | 993.5115 | y9 | 21.1 | 260 |
|  |  |  | 854.4573 |  | 846.4431 | y8 | 19.1 | 260 |
|  |  |  | 755.3889 |  | 747.3747 | y7 | 18.1 | 260 |
|  | SAAGNASSTFYTDK | 714.3301 | 956.2000 | 710.3230 | 948.1858 | y8 | 15.7 | 100 |
|  |  |  | 1027.4000 |  | 1019.3858 | y9 | 15.7 | 100 |
|  |  |  | 869.2000 |  | 861.1858 | y7 | 15.7 | 100 |
|  |  |  | 782.2000 |  | 774.1858 | y6 | 15.7 | 100 |
|  | DLVSNYTIDAIR | 695.3660 | 1062.4000 | 690.3619 | 1052.3917 | y9 | 14.6 | 260 |
|  |  |  | 861.2000 |  | 851.1917 | y7 | 14.6 | 260 |
|  |  |  | 975.4000 |  | 965.3917 | y8 | 14.6 | 260 |
|  |  |  | 1161.6000 |  | 1151.5173 | y10 | 14.6 | 260 |
|  |  |  | 597.0000 |  | 586.9173 | y5 | 14.6 | 260 |
|  | VLIEDSQK | 470.2655 | 614.2000 | 466.2584 | 606.1858 | y5 | 3.2 | 280 |
|  |  |  | 727.2000 |  | 719.1858 | y6 | 3.2 | 280 |
|  |  |  | 840.2000 |  | 832.1858 | y7 | 3.2 | 280 |
| Glyoxal oxidase | IIMVGSGK | 406.7430 | 699.3549 | 402.7360 | 691.3407 | y7 | 8.0 | 220 |
| (CNAG_00407) |  |  | 586.2709 |  | 578.2567 | y6 | 8.0 | 220 |
|  |  |  | 455.2304 |  | 447.2162 | y5 | 8.0 | 220 |
|  |  |  | 356.1620 |  | 348.1478 | y4 | 9.0 | 220 |
|  | YLELNSTYTK | 620.3210 | 1076.5214 | 616.3139 | 1068.5072 | y9 | 18.9 | 260 |
|  |  |  | 963.4373 |  | 955.4231 | y8 | 16.9 | 260 |
|  |  |  | 834.3947 |  | 826.3805 | y7 | 16.9 | 260 |
|  |  |  | 721.3106 |  | 713.2964 | y6 | 15.9 | 260 |
|  | AFSQQQAR | 473.2420 | 874.3905 | 468.2383 | 864.3822 | y7 | 11.3 | 220 |
|  |  |  | 640.2901 |  | 630.2818 | y6 | 11.3 | 220 |
|  |  |  | 512.2315 |  | 502.2232 | y5 | 9.3 | 220 |
|  |  |  | 384.1729 |  | 374.1646 | y4 | 11.3 | 220 |
|  | DVTFEK | 373.6942 | 431.0000 | 369.6871 | 422.9858 | y3 | 0.3 | 260 |
|  |  |  | 532.2000 |  | 524.1858 | y4 | 0.3 | 260 |
|  |  |  | 623.0000 |  | 614.9858 | y5 | 0.3 | 260 |
|  | EGLGMTTQER | 566.2705 | 644.2000 | 561.2664 | 634.1917 | y5 | 8.1 | 220 |
|  |  |  | 775.2000 |  | 765.1917 | y6 | 8.1 | 220 |
|  |  |  | 832.2000 |  | 822.1917 | y7 | 8.1 | 220 |
|  |  |  | 442.0000 |  | 431.9917 | y3 | 8.1 | 220 |
|  | EGLGMTTQER | 582.2654 | 525.0000 | 577.2613 | 514.9917 | y4 | 8.1 | 240 |
|  | modification: oxidation |  | 644.0000 |  | 633.9917 | y5 | 8.1 | 240 |
|  |  |  | 806.8000 |  | 796.7917 | y6 | 8.1 | 240 |
|  |  |  | 864.2000 |  | 854.1917 | y7 | 8.1 | 240 |
| Acid phosphatase | GDLDFLNK | 465.2440 | 757.3834 | 461.2374 | 749.3692 | y6 | 8.0 | 220 |
| (CNAG_02944) |  |  | 644.2994 |  | 636.2852 | y5 | 7.0 | 220 |
|  |  |  | 529.2724 |  | 521.2582 | y4 | 11.0 | 220 |
|  |  |  | 382.2040 |  | 374.1898 | y3 | 11.0 | 220 |
|  | GFLEEFVAR | 539.2840 | 873.4204 | 534.2796 | 863.4121 | y7 | 14.7 | 240 |
|  |  |  | 760.3363 |  | 750.3281 | y6 | 14.7 | 240 |
|  |  |  | 631.2938 |  | 621.2855 | y5 | 12.7 | 240 |
|  |  |  | 502.2512 |  | 492.2429 | y4 | 14.7 | 240 |
|  | LGAELLTPFGR | 592.3390 | 1070.5368 | 587.3350 | 1060.5286 | y10 | 17.4 | 260 |
|  |  |  | 942.4783 |  | 932.4700 | y8 | 17.4 | 260 |
|  |  |  | 813.4357 |  | 803.4274 | y7 | 17.4 | 260 |
|  |  |  | 700.3516 |  | 690.3433 | y6 | 17.4 | 260 |
|  |  |  | 486.2199 |  | 476.2116 | y4 | 13.4 | 260 |
|  | LQNFELGVTFR | 667.3605 | 702.4000 | 662.3564 | 692.3917 | y6 | 13.2 | 60 |
|  |  |  | 831.4000 |  | 821.3917 | y7 | 13.2 | 60 |
|  |  |  | 1092.8000 |  | 1082.7917 | y9 | 13.2 | 60 |
| Hypothetical | IGNVEQIVVSYCLK | 815.4416 | 876.2000 | 811.4345 | 868.1858 | y7 | 20.8 | 60 |
| (CNAG_05312) | modification: carbamidation |  | 989.4000 |  | 981.3858 | y8 | 20.8 | 60 |
|  |  |  | 1117.4000 |  | 1109.3858 | y9 | 20.8 | 60 |
|  |  |  | 777.0000 |  | 768.9858 | y6 | 20.8 | 60 |
|  |  |  | 1246.2000 |  | 1238.1858 | y10 | 20.8 | 60 |
|  | VSYVQVTGVGDLTK | 737.4056 | 798.2000 | 733.3985 | 790.1858 | y8 | 16.8 | 60 |
|  |  |  | 897.2000 |  | 889.1858 | y9 | 16.8 | 60 |
|  |  |  | 1025.6000 |  | 1017.5858 | y10 | 16.8 | 60 |
|  |  |  | 697.2000 |  | 689.1858 | y7 | 16.8 | 60 |
|  |  |  | 540.8000 |  | 532.7858 | y5 | 16.8 | 60 |
|  | VIPPGAITGAHFVK | 707.9185 | 601.6000 | 703.9114 | 593.5858 | y5 | 15.3 | 280 |
|  |  |  | 475.2000 |  | 467.1858 | y4 | 15.3 | 280 |
|  |  |  | 949.2000 |  | 941.1858 | y9 | 15.3 | 280 |
|  |  |  | 1105.4000 |  | 1097.3858 | y11 | 15.3 | 280 |
|  |  | 472.2814 | 601.8000 | 469.6100 | 593.7858 | y5 | 2.6 | 180 |
|  |  |  | 802.4000 |  | 794.3858 | y7 | 2.6 | 180 |
|  | NGYGTR | 339.1656 | 506.0000 | 334.1615 | 495.9917 | y4 | 2.5 | 80 |
|  |  |  | 563.2000 |  | 553.1917 | y5 | 2.5 | 80 |

^a^For the majority of peptides, at least 3 transition ions were monitored for quantitation and confirmation.

^b^Collision energy

^c^Fragmentor voltage
